# Galactooligosaccharide Treatment Alleviates DSS-Induced Colonic Inflammation in Caco-2 Cell Model

**DOI:** 10.3389/fnut.2022.862974

**Published:** 2022-04-14

**Authors:** Marianna Roselli, Aleksandra Maruszak, Roberta Grimaldi, Lucien Harthoorn, Alberto Finamore

**Affiliations:** ^1^Research Centre for Food and Nutrition, CREA (Consiglio per la ricerca in agricoltura e l'analisi dell'economia agraria), Rome, Italy; ^2^Clasado Biosciences Ltd., Reading, United Kingdom

**Keywords:** Inflammatory Bowel Disease, ulcerative colitis, prebiotic, galactooligosaccharides, cytokines, gut barrier, gut inflammation

## Abstract

The biological activities of dietary bioactive polysaccharides have been largely explored. Studies on the immunomodulating effects of oligosaccharides and polysaccharides have shown that they are able to modulate innate immunity. Prebiotics are a class of poorly digested carbohydrates that are mainly produced from dietary fibers, which are carbohydrate polymers with ten or more monomeric units as defined by the Codex Alimentarius Commission in 2009. Considering the capacity of prebiotics in reducing gut inflammation, the aim of this study was to investigate the anti-inflammatory activity of galactooligosaccharide (Bimuno^®^ GOS) in an *in vitro* model of ulcerative colitis (UC)-like inflamed intestinal cells. Differentiated Caco-2 cells were exposed to 2 % dextran-sulfate-sodium salt (DSS) to induce inflammation, and then with different concentrations of Bimuno GOS (1–1,000 μg/ml). Cell monolayer permeability, tight- and adherent junction protein distribution, pro-inflammatory cytokine secretion, and NF-kB cascade were assessed. Bimuno GOS at different concentrations, while not affecting cell monolayer permeability, was shown to counteract UC-like intestinal inflammatory responses and damages induced by DSS. Indeed, Bimuno GOS was able to counteract the detrimental effects of DSS on cell permeability, determined by transepithelial electrical resistance, phenol red apparent permeability, and tight- and adherent junction protein distribution. Furthermore, Bimuno GOS inhibited the DSS-induced NF-kB nuclear translocation and pro-inflammatory cytokine secretion. Further analyses showed that Bimuno GOS was able to revert the expression levels of most of the proteins involved in the NF-kB cascade to control levels. Thus, the prebiotic Bimuno GOS can be a safe and effective way to modulate the gut inflammatory state through NF-kB pathway modulation, and could possibly further improve efficacy in inducing remission of UC.

## Introduction

Inflammatory Bowel Disease (IBD), including Crohn's disease (CD) and ulcerative colitis (UC), are chronic inflammatory disorders characterized by a progressive disease course involving the gastrointestinal tract. Traditionally, IBD has been considered as a disease of the Western world, however, data from the last 10 years describe a continuous increase of its incidence in newly industrialized geographies such as Asia and Latin America (1, 2). The etiology of UC is still not completely known, multiple factors such as genetic background, environmental factors, microbiota, and mucosal immune dysregulation seem to be involved in its onset (3, 4). UC generally affects the colon and rectum with a relapsing and remitting pattern. It is an idiopathic intestinal inflammatory disease presenting typical symptoms (3), such as rectal bleeding, bloody and mucous diarrhea, and abdominal pain, however, the presence of extra-intestinal manifestations is also known and used for the diagnosis of UC (5). UC characteristic pathophysiology involves epithelial barrier and increased uptake of luminal antigens caused by defects in colonic mucin secretion and tight junctions damages (6). An increased number of activated and mature dendritic cells with a high expression of Toll-Like Receptor (TLR)2 and TLR4 has been observed in the lamina propria of patients affected by IBD (7), and loss of negative regulation of TLR signaling has been associated with the disruption of intestinal homeostasis (8). An atypical T-helper (Th)-cell response, as well as a specific pro-inflammatory cytokine pattern, have been shown to exist in patients affected by UC (9). Conventional therapies consist of pharmacologic agents including aminosalicylates and corticosteroids, which are able to induce remission and mucosal healing and to prevent relapses to avoid colectomy in patients affected by UC. New pharmacological strategies such as monoclonal antibodies targeted to specific pro-inflammatory cytokines, adhesion molecules, T-cell activation, as well as agents inducing anti-inflammatory cytokines [i.e., IL-10, transforming growth factor (TGF)-β], have been also used, however, they are often associated with adverse effects including headache, nausea, abdominal pain and cramping, loss of appetite, vomiting and rash (10).

Clinical and animal studies indicate that UC pathophysiology is strongly associated with changes in the gut microbiota composition (11, 12) including reduced species richness (13). Microbiome dysbiosis in UC plays a critical role in the innate intestinal immunity, however, whether these changes are a cause, or a consequence of the disease remains to be elucidated (14). Overall, considering the close connection between intestinal microbiota and UC pathogenesis, several studies have shown that intestinal dysbiosis contributes to the pathogenesis of this disease (13).

In animal models, experimental colitis is commonly induced by dextran sodium sulfate (DSS) dissolved in drinking water. Rodents treated with DSS exhibit clinical features very similar to those observed in humans affected by UC, including increased intestinal mucosal permeability and gut inflammation characterized by the secretion of specific cytokines (15). Moreover *in vitro* models of intestinal cells treated with DSS are also used to investigate mechanisms associated with UC pathogenesis (16–18). In particular, it has been shown that DSS alters Caco-2 cell tight junctions, cell cycle metabolism, as well as cytokine release (18). DSS in Caco-2 cells leads to an increased pro-inflammatory cytokine expression pattern (IL-2, IL-8, TNF-α) and gut barrier damages characterized by loss of zonula occludens-1 (ZO-1), occludin, and claudin-1, that are similar to the changes observed in *in vivo* models and patients with UC (19).

Considering that on one hand, IBD patients have been shown to have dysbiosis and reduced microbiota diversity, and on the other hand, a relationship between UC and gut microbiota has been observed, several studies investigated if microbiota modulation can ameliorate UC symptoms. There is growing evidence that probiotic and prebiotic supplementation can positively modulate gut microbiota composition by inducing microbiota restitution, which is fundamental to ensure gut health maintenance and immune homeostasis in UC patients.

Prebiotics are defined as “a substrate that is selectively utilized by host microorganisms conferring a health benefit” (20). Prebiotics are a substrate for beneficial gut bacteria able to produce short-chain fatty acids (SCFAs), which have been shown to play a key role in the suppression of inflammation in IBD (21). Thus, prebiotics or fiber-rich diets lead to an increase in SCFAs-producing microbiota.

Prebiotics include galactooligosaccharides (GOS), fructo-oligosaccharides (FOS), inulin, and β-glucans (22). The potential use of prebiotics in IBD has been suggested since they are able to promote probiotic microorganisms' growth and stimulate innate immunity (23). GOS is a term indicating a group of carbohydrates composed of oligo-galactose with lactose molecules and glucose monomers (24). The products of lactose extension are classified into two smaller groups, the GOS with excess galactose at C3, C4, or C6 and the GOS manufactured from lactose through enzymatic trans-glycosylation (25). A recent study demonstrated normalization of stools and a reduction of the incidence and severity of loose stools alongside lower urgency and a specific prebiotic effect by GOS in a cohort of patients with UC (26). Some preclinical and clinical studies highlighted that prebiotics may exert beneficial effects on UC symptoms (27) including a reduction of gut inflammation (28), bloating, diarrhea, constipation, abdominal pain, fecal pH reduction, as well as promoting regular intestinal peristalsis and preventing the mucus layer degradation (27). It has been recognized that one of the SCFA, butyrate, is associated with decreased inflammation and may alleviate UC symptoms (29). For the above-described reasons, prebiotics can be considered a valid alternative to conventional UC therapies.

A reduction of markers of intestinal inflammation, such as fecal calprotectin, has also been observed in patients with UC with active disease after supplementation with inulin (30). Experimental evidence suggests that GOS are metabolized by bacteria that possess β-galactosidases, such as Bifidobacterium species (31, 32). In particular, the major bifidogenic effect has been observed with a specific GOS (Bimuno GOS) (33, 34). This GOS has an important role in the immune function modulation, as observed in a study on a population of elderly supplemented with the GOS, where it induced IL-10 increase and reduction of IL-1β. This specific GOS has also been shown to reduce serum IL-8, C-Reactive Protein (CRP), and to improve NK cell activity (34).

In *in vitro* models, GOS has also been shown to modulate the epithelial barrier function by inducing differentiation and epithelial wound repair, and by promoting the growth of specific gut bacteria, associated with changes in SCFA profiles (35). The effect of GOS on epithelial cells has been further confirmed by a transcriptomic analysis performed on Caco-2 cells, showing that GOS was able to modulate the expression of several genes implicated in digestion and transepithelial transport, which contribute to intestinal cell integrity and function (36).

Several studies have suggested that prebiotics, in addition to the ability to modulate the intestinal microbiota, can also exert a direct action on the intestinal epithelium through the induction of an anti-inflammatory response (37–39). In the present study, we aimed to analyze the direct effect of a specific GOS (Bimuno) in an *in vitro* model of UC-like inflammation. We treated the intestinal Caco-2 cells with DSS to mimic the inflammatory state present in the intestinal mucosa of UC patients, and we identified the possible mechanisms associated with GOS treatment for improvement of the inflammatory status in the context of UC.

## Materials and Methods

### Epithelial Cell Culture

The Caco-2/TC7 cells, a clone derived from parental human intestinal Caco-2 cell line at late passage, were a kind gift from Dr. Monique Rousset (Institute National de la Santé et de la Recherche Médicale, INSERM, France). These cells are characterized by a more homogeneous expression of differentiation traits with more developed intercellular junctions and have been observed to exhibit higher metabolic and transport activities, being more similar to enterocytes of the small intestine than the original cell line (40).

Caco-2/TC7 cells were maintained at 37°C in an atmosphere of 5% CO_2_/95% air at 90% relative humidity on plastic tissue culture flasks (75 cm^2^ growth area, Becton Dickinson, Milan, Italy), in Dulbecco's modified minimum essential medium (DMEM; 3.7 g/L NaHCO_3_), supplemented with 4 mm glutamine, 10% heat-inactivated fetal calf serum, 1% non-essential amino acids, 10^5^ U/L penicillin, and 100 mg/L streptomycin. The cell culture media and reagents were purchased from Euroclone (Milan, Italy). The cells were used between 80 and 105 passages. For the experimental assays, the cells were seeded at a density of 1 × 10^6^ cells/filter on polyethylene terephthalate semipermeable filters (Transwell^®^, 12 mm diameter,0.45 μm pore size, Becton Dickinson), which allow epithelial differentiation between apical (AP) and basolateral (BL) compartments. After reaching confluency, the cells were left for 17–21 days to allow full differentiation. The medium was changed 3 times a week. To induce inflammation, differentiated Caco-2/TC7 cells were exposed to dextran sulfate sodium salt (DSS, MW: 40.000; Sigma, Milan, Italy). Preliminary experiments were performed to choose the optimal DSS concentration in the 0.05–5 % range, as well as the suitable time of treatment.

### Cell Permeability Assessments

Cell membrane permeability was assayed by measuring the transepithelial electrical resistance (TEER), according to Ferruzza et al. (41). TEER was monitored every day until differentiation using a Millicell Electrical Resistance system (Merck Millipore, Darmstadt, Germany), and expressed as Ohm (resistance) × cm^2^ (surface area of the filter), after subtracting the resistance value of the filter without cell monolayer. The TEER was checked before each experimental assay, and only cell monolayers with TEER values higher than 1,000 Ohm × cm^2^ were used, as this TEER value was identified in preliminary experiments as indicative of correct differentiation in Caco-2/TC7 cells. During the experiments, TEERs were recorded every 30–60 min. Cell permeability was also measured at the end of treatments by measuring phenol red passage, as reported by Ferruzza et al. (41). Briefly, after three washes with phosphate buffered saline (PBS) containing Ca^++^ and Mg^++^ (PBS^++^), 0.5 ml 1 mm phenol red was added to the AP compartment of cell monolayers, whereas 1 ml PBS^++^ was added in the BL compartment. After 1 h incubation at 37°C, 0.9 ml, the BL medium was collected, added with 0.1 ml 0.1 N NaOH, and read at 560 nm to determine the phenol red concentration (Tecan Infinite M200 microplate reader, Tecan Italia, Milan, Italy). This concentration was used to calculate the phenol red apparent permeability coefficient (Papp) by applying the following formula: Papp = Ct x V_BL_/Δt x·C_0_ × A, where V_BL_ is the volume of the BL compartment (cm^3^), A is the filter area (cm^2^), Δt is the time interval (s), Ct is the phenol red concentration in the BL compartment at the end of time interval, and C_0_ is the phenol red concentration in the AP compartment at the beginning. The tight junctions were considered open and indicative of an absence of cell monolayer integrity when the phenol red Papp values were above 1 × 10^−6^ cm/s, as evaluated by previous reports in the literature (42). Differences observed among samples with such values of phenol red Papp were thus considered biologically irrelevant, irrespective of statistical significance.

### GOS Preparation

Bimuno^®^ in powder form with a content of 80% active GOS, 14% lactose, 5% glucose, and ~1% galactose on dry matter, was supplied by Clasado Biosciences Ltd. (Reading, UK). The composition of the active GOS in terms of the degree of polymerization (DP), which refers to the number of monomeric units, was as follows: DP2 25%, DP3 41%, DP4 20%, DP5 9%, and DP>5 5%. Final concentrations of active GOS were made from 1 to 1,000 μg/ml by dissolving it as a powder in a serum free-cell culture medium.

### Cell Toxicity of GOS

Several GOS concentrations were tested on Caco-2/TC7 cells differentiated on Transwell filters to assay the potential toxicity, by measuring TEER every 60 min for 24 h and phenol red Papp at the end of treatment. The concentrations tested ranged from 1 to 1,000 μg/ml (calculated from the percentage of active GOS). The cell monolayers were kept in the serum-free medium overnight before the experiments, to avoid possible interferences with serum proteins.

### Inflammation Induction by DSS

Dextran-sulfate-sodium salt was used to induce inflammation in differentiated Caco-2/TC7 cells. In order to identify the suitable concentration of DSS to activate inflammatory status without inducing serious damages to cell monolayers, different concentrations of DSS, ranging from 0.05 to 5 % were tested for 5.5 h, and TEER, phenol red Papp, and induction of NF-kB activation were measured.

### Cell Treatment With DSS and Bimuno GOS

**Figure 1:**
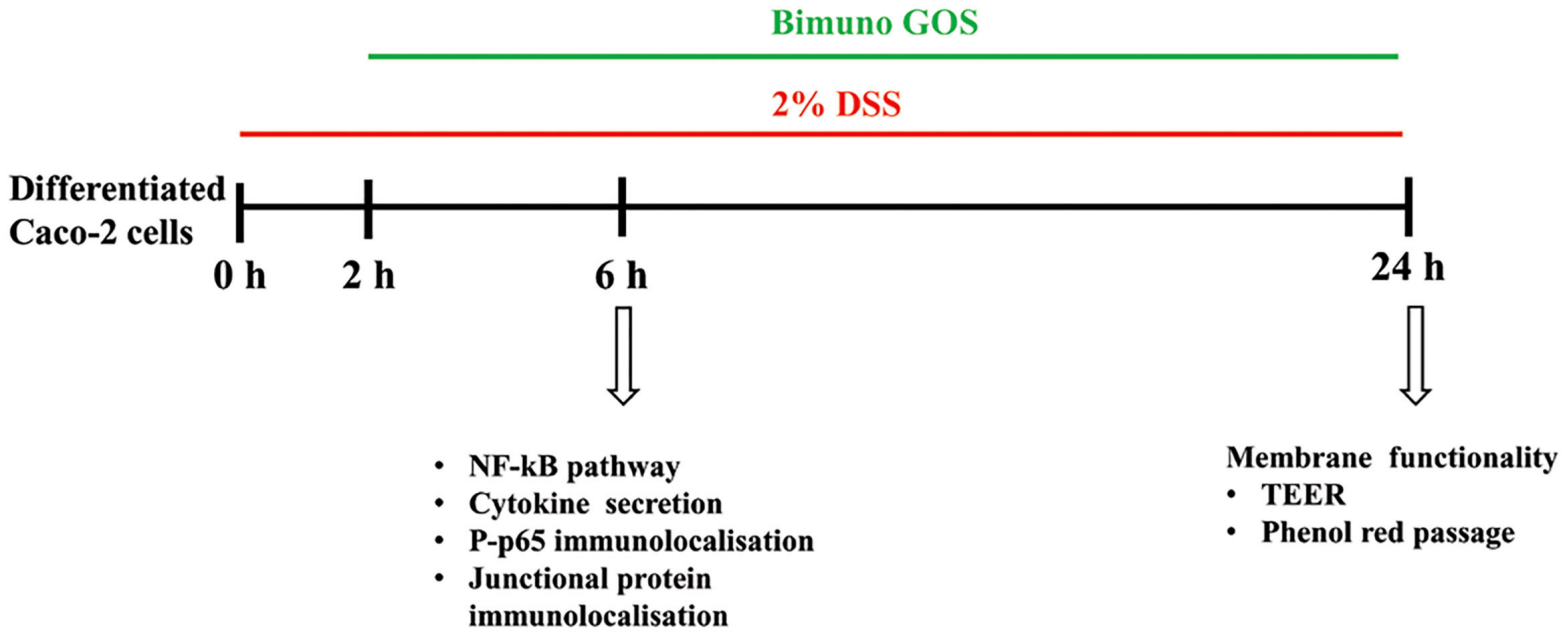
Experimental design. Three different experimental setups were employed, as differentiated Caco-2 cells were: (1) treated with 2% dextran-sulfate-sodium salt (DSS) alone (6 or 24 h), (2) treated with 2% DSS for 2 h, then several Bimuno galactooligosaccharides (GOS) concentrations (1–1,000 μg/ml) were added for further 4 or 22 h, or (3) treated with different Bimuno GOS concentrations alone (4 or 20 h). The different analyses performed at each time point are indicated.

There were three different experimental setups: differentiated Caco-2/TC7 cells were either: 1-untreated cells (Blank Control; C), 2- treated with 2% DSS alone (6 h, positive control; DSS), 3-treated with 2 % DSS for 2 h, then several concentrations of Bimuno GOS (1–1,000 μg/ml) were added for further 4 h, or 4- treated with different Bimuno GOS concentrations alone (4 h). The different time points, according to the different experiments performed, are shown in Figure 1.

### Western Blot Assay of TLR4 Signaling Proteins

Differentiated Caco-2/TC7 cells were treated according to the three different experimental setups, with GOS concentrations 100 or 200 μg/ml. At the end of treatments, the cells were washed with cold PBS and lysed in a cold radioimmunoprotein assay buffer (RIPA: 20 mM Tris-HCl pH 7.5, 150 mM NaCl, 0.1% SDS, 1% Na deoxy-cholate, 1% Triton X-100) supplemented with 1 mm phenylmethylsulphonyl fluoride, and protease inhibitor (Complete Mini, Roche, Milan, Italy) and phosphatase inhibitor (PhosSTOP, Roche) cocktails, according to Finamore et al. (43). Cell lysates (50 μg total proteins) were dissolved in sample buffer (50 mM Tris-HCl pH 6.8, 2 % SDS, 10 % glycerol, 100 g/L bromophenol blue, 10 mm β-mercaptoethanol), heated for 5 min, fractionated by SDS-polyacrylamide gel (4–20% gradient) electrophoresis and transferred to nitrocellulose filters (Trans-Blot Turbo, Biorad, Milan, Italy). Membranes were incubated with the following primary antibodies: rabbit polyclonal anti-human TLR4, MyD88, IKKα, IKKβ, phospho(P)-IKKα/β, IkBα, P-IkBα, NF-kB p65, P-p65, IRAK-M, Tollip, from Cell Signaling Technology (Danvers, MA), mouse monoclonal α-tubulin. Proteins were detected with horseradish peroxidase-conjugated secondary antibodies (Cell Signaling Technology) and enhanced chemiluminescence reagent (ECL kit Lite Ablot Extend, Euroclone), followed by the analysis of chemiluminescence with the charge-coupled device camera detection system Las4000 Image Quant (GE Health Care Europe GmbH, Milan, Italy). Relative levels of TLR4, MyD88, Tollip, and IRAK-M were normalized to α-tubulin, whereas the phosphorylated proteins were normalized to their corresponding unphosphorylated forms.

### Cytokine Secretion

Differentiated Caco-2/TC7 cells were treated according to the three different experimental setups, with GOS concentrations 1–200 μg/ml. Secretion of pro-inflammatory cytokines IL-1β, IL-6, IL-8. and TNF-α was measured by ELISA (Biolegend, San Diego, CA) in the cell supernatants collected from the AP compartments at the end of treatments, following the manufacturer's instruction. Supernatants were centrifuged at 650 × g for 5 minin at 4°C to remove cell debris, aliquoted, and immediately frozen at −80°C. In the preliminary experiments, cytokines were measured also in the culture media collected from the (BL) compartment, but results showed undetectable levels (data not shown).

### Localization of TJ (ZO-1 and Occludin) Proteins, AJ (E-Cadherin and β-Catenin) Proteins, and P-P65

Differentiated Caco-2/TC7 cells were treated according to the three different experimental setups, with GOS concentrations 100 or 200 μg/ml. The effect of GOS on membrane damage induced by DSS was assessed by evaluating tight and adherent junctions' principal proteins immunolocalization, as well as P-p65 immunolocalization. Briefly, at the end of the experiments, Caco-2/TC7 cells were washed with cold PBS++, fixed in ice-cold methanol for 3 mins, and then incubated with rabbit polyclonal anti-ZO-1 and mouse monoclonal anti-occludin, or mouse monoclonal anti-β-catenin and rabbit polyclonal anti-E-cadherin antibodies (Zymed Laboratories, San Francisco, CA), or rabbit polyclonal anti-P-p65 antibody (Cell Signaling Technology, Danvers, MA) for 1 h. For secondary detection, cells were incubated with fluorescein isothiocyanate (FITC) or tetramethylrhodamine isothiocyanate (TRITC) conjugated secondary antibodies (Jackson Immunoresearch, Milan, Italy), for 1 h. 4′,6-diamidino-2-phenylindole (DAPI) was used to label DNA in nuclei. Stained monolayers were mounted on glass slides using the Prolong Gold antifade reagent (Molecular Probes, Invitrogen, Milan, Italy) and analyzed using a confocal fluorescence microscope (LSM 700, Zeiss, Jena, Germany).

### Statistical Analysis

All experiments were performed at least in triplicate. The statistical significance of the differences was evaluated by one-way ANOVA followed by a *post-hoc* Tukey HSD test, after verifying the normality and homogeneity of variance by Shapiro–Wilk's and Levene's tests, respectively. Statistical significance was set at *P* < 0.005. In the figures, mean values with different superscript letters significantly differ. *P* < 0.01 or 0.001 are indicated, where appropriate. In TEER figures, only final and initial time points were compared for statistical analysis. The statistical analyses were executed with the “Statistica” software package (version 5.; StatSoftInc., Tulsa, OK, USA).

## Results

The different experimental setups and different timepoints are shown in Figure 1.

### Effect of GOS and DSS on Caco-2/TC7 Cell Permeability

**Figure 2:**
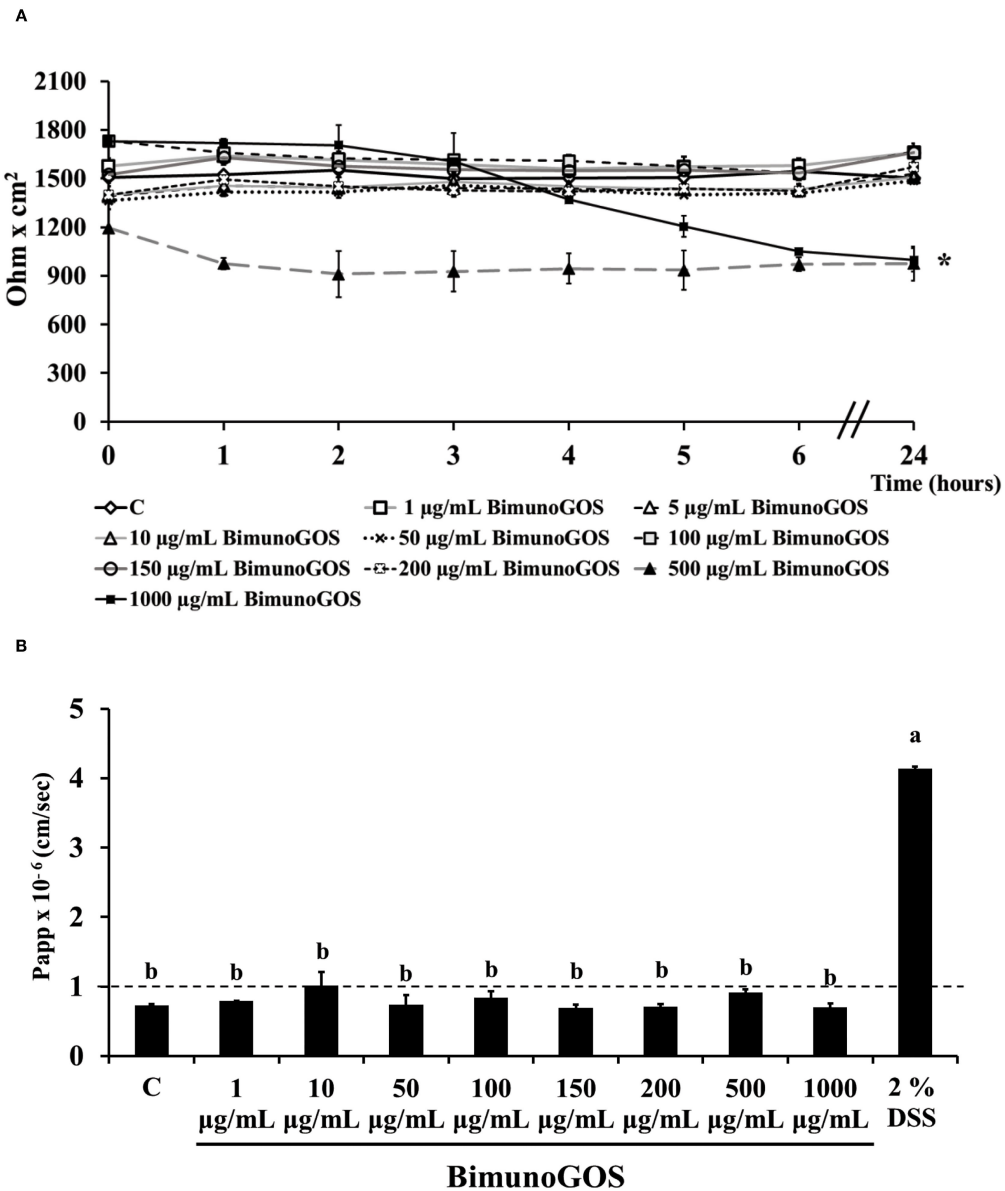
Bimuno GOS effects on membrane barrier of Caco-2 cells. Transepithelial electrical resistance (TEER) **(A)** and phenol red apparent permeability (Papp) **(B)**. Cells were untreated (Control, C), treated with different Bimuno GOS concentrations (1–1,000 μg/ml), or treated with 2% DSS. TEER values are reported as Ohm × cm^2^. Phenol red Papp was measured after 24 h treatment with Bimuno GOS or after 6 h treatment with DSS, and values are reported as cm/s. Values represent mean ± SD of at least three independent experiments, carried out in triplicate. **(A)** *Stands for significant difference between T0 and T24 (*P* < 0.001). **(B)** Means without a common letter significantly differ, *P* < 0.001.

Preliminary experiments were performed to evaluate the toxicity of GOS on Caco-2 cells. TEER and phenol red Papp were measured in differentiated Caco-2 cells after treatment with several GOS concentrations for up to 24 h. The results show that GOS treatment did not affect cell permeability, except for the highest GOS concentration, which induced a TEER drop after 3 h, which was maintained until the end of the experiment (Figure 2A). However, this TEER decrease was not associated with a biologically relevant phenol red Papp increase, as the values were all below 1 × 10^−6^ cm/s, indicating that the tight junctions were not open. On the contrary 2% DSS treatment induced a strong increase on phenol red passage (*P* < 0.001, Figure 2B; Supplementary Table S1).

**Figure 3:**
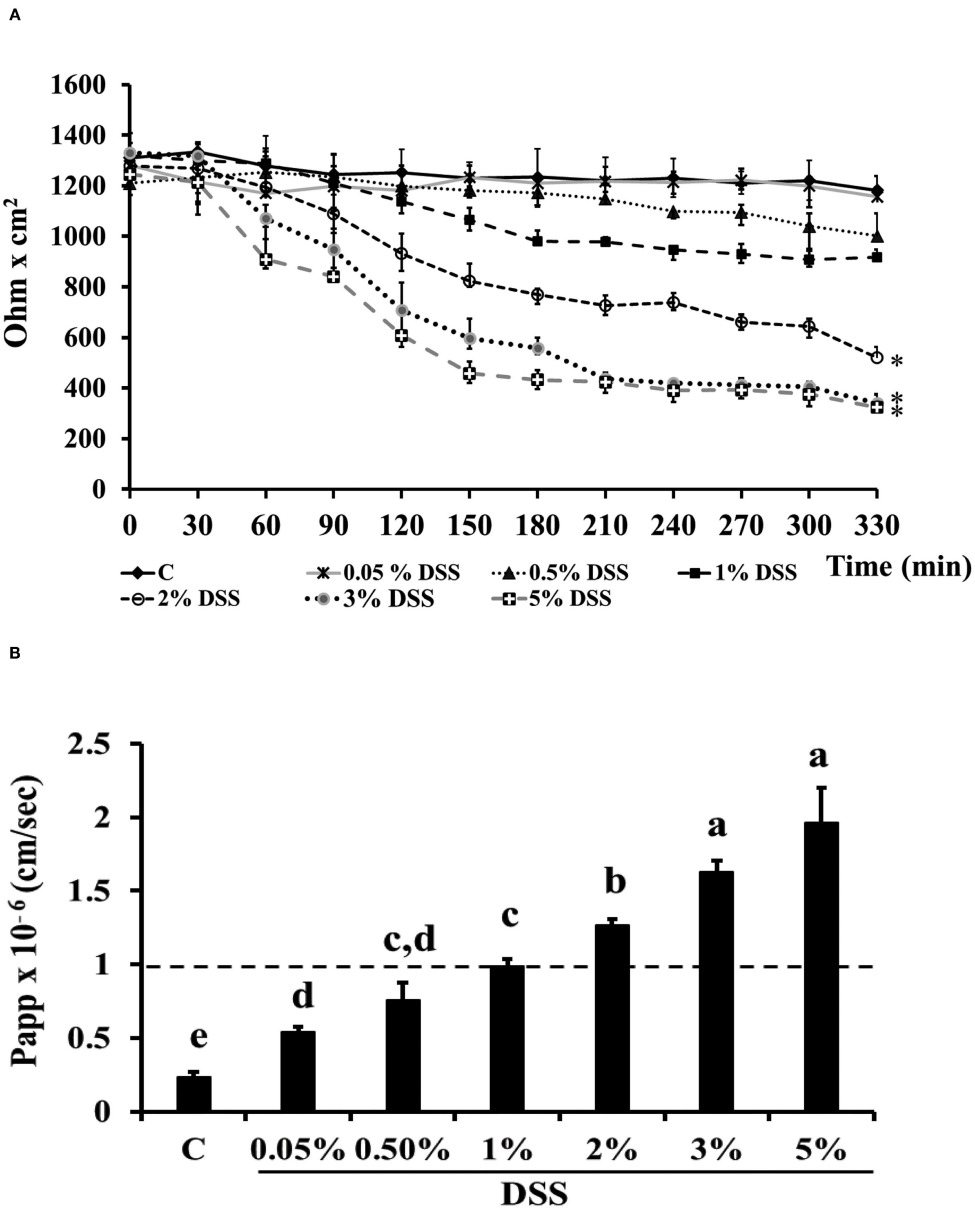
DSS effects on membrane barrier of Caco-2 cells. Transepithelial electrical resistance (TEER) **(A)** and phenol red apparent permeability (Papp) **(B)**. Cells were either untreated (Control, C) or treated with different DSS concentrations (0.05–5%) for 330 min. TEER values are reported as Ohm × cm^2^. Phenol red Papp was measured at end of TEER measurements, and values are reported as cm/sec. Values represent mean ± SD of at least three independent experiments, carried out in triplicate. **(A)** *Stands for significant difference between T0 and T330 (*P* < 0.001). **(B)** Means without a common letter significantly differ, *P* < 0.05.

Dextran-sulfate-sodium salt was used to induce inflammation in differentiated Caco-2 cells. In order to determine the DSS concentration able to trigger inflammation without inducing serious damages to the cell monolayer, different DSS concentrations were tested for 5.5 h, by measuring TEER and phenol red passage (Figures 3A,B). Treatment with DSS concentrations ranging between 0.05 and 1% did not alter cell permeability, as shown by both TEER measurements and phenol red Papp (Figures 3A,B, respectively). On the contrary, 3 and 5% DSS induced a dramatic TEER drop starting from 2.5 h until 5.5 h (Figure 3A), which was accompanied by a phenol red Papp higher than 1 × 10^−6^ cm/s (Figure 3B). The 2% DSS induced a lower TEER drop as compared to 3 and 5% (Figure 3A), and a slight increase of phenol red passage, borderline with the threshold value of 1 × 10^−6^ cm/s (Figure 3B; Supplementary Table S2), thus this concentration was chosen for further experiments, as supposed to be able to induce inflammation, without seriously damaging cell monolayers.

### GOS Exerted a Protective Effect Against DSS-Induced Membrane Barrier Damage

**Figure 4:**
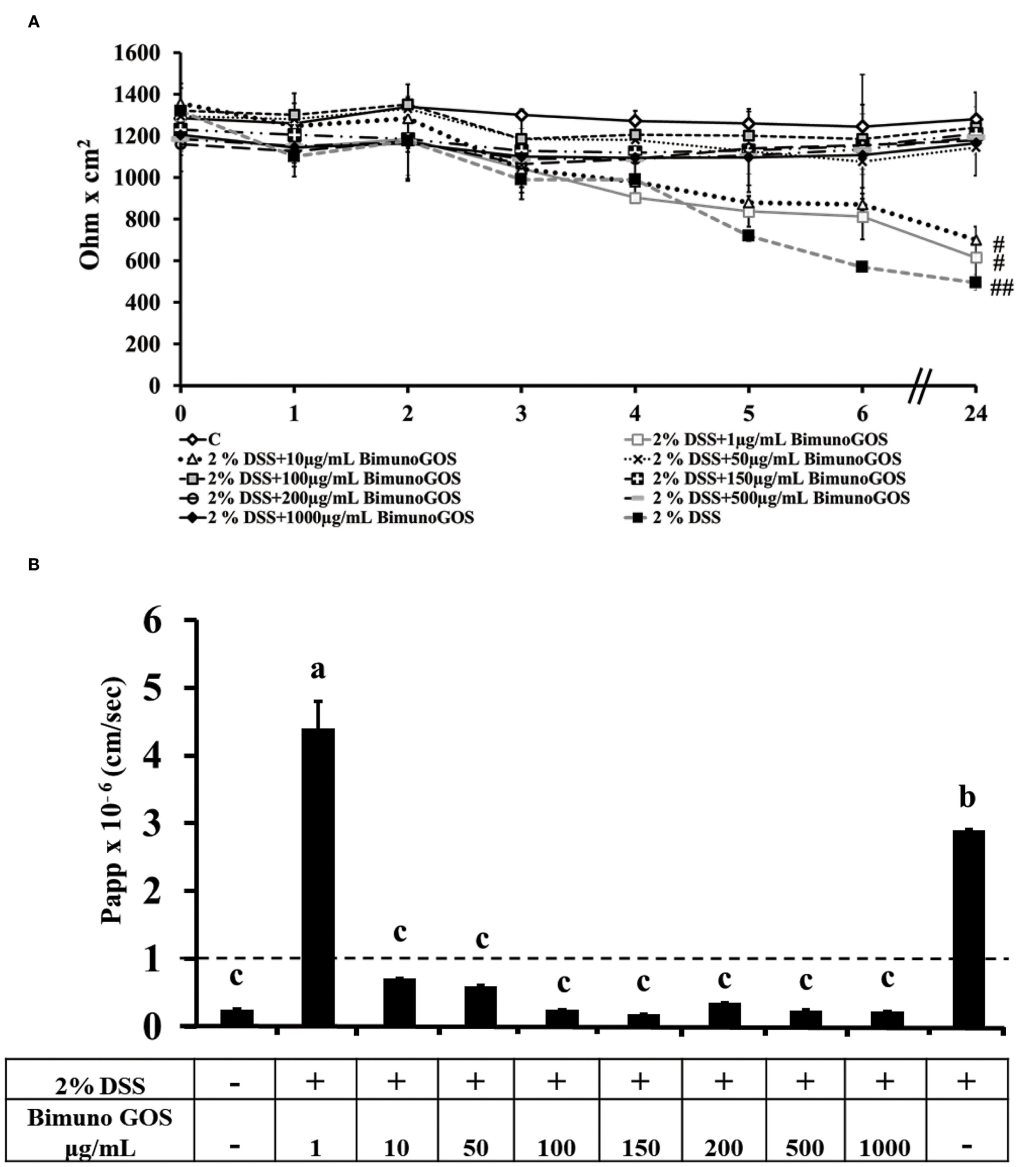
Protective effect of Bimuno GOS against membrane barrier damages induced by DSS in Caco-2 cells. Transepithelial electrical resistance (TEER) **(A)** and phenol red apparent permeability (Papp) **(B)**. Cells were either untreated (C) or treated with 2% DSS for 2 h and then with several Bimuno GOS concentrations (1–1,000 μg/ml) up to 24 h. TEER values are reported as Ohm × cm^2^. Phenol red Papp values are reported as cm/s. Values represent mean ± SD of at least three independent experiments, carried out in triplicate. **(A)** ^#^,^##^Stand for significant difference between T0 and T24 (*P* < 0.01 and *P* < 0.001, respectively). Values represent mean ± SD of at least three independent experiments, carried out in triplicate. **(B)** Means without a common letter significantly differ, *P* < 0.05.

To induce inflammation differentiated Caco-2 cells were pre-treated with 2 % DSS for 2 h, then GOS was added for additional 4 h, to test its protective effect. TEER analysis showed that GOS concentrations ranging from 50 to 1,000 μg/ml were able to prevent the TEER decrease induced by DSS, whereas 1 and 10 μg/ml concentrations only partially protected the cells from the DSS-induced TEER drop (Figure 4A). Analysis of phenol red Papp showed that all GOS concentrations were able to protect the cells from DSS damage, except for the lowest one (1 μg/ml, Figure 4B; Supplementary Table S3). Based on these data, the 100 and 200 μg/ml GOS concentrations were chosen for further experiments, considering that these concentrations were not detrimental for cell monolayers and were able to protect cells against DSS-induced cell damages.

### GOS Reduced the DSS-Induced Pro-Inflammatory Cytokine Secretion

**Figure 5:**
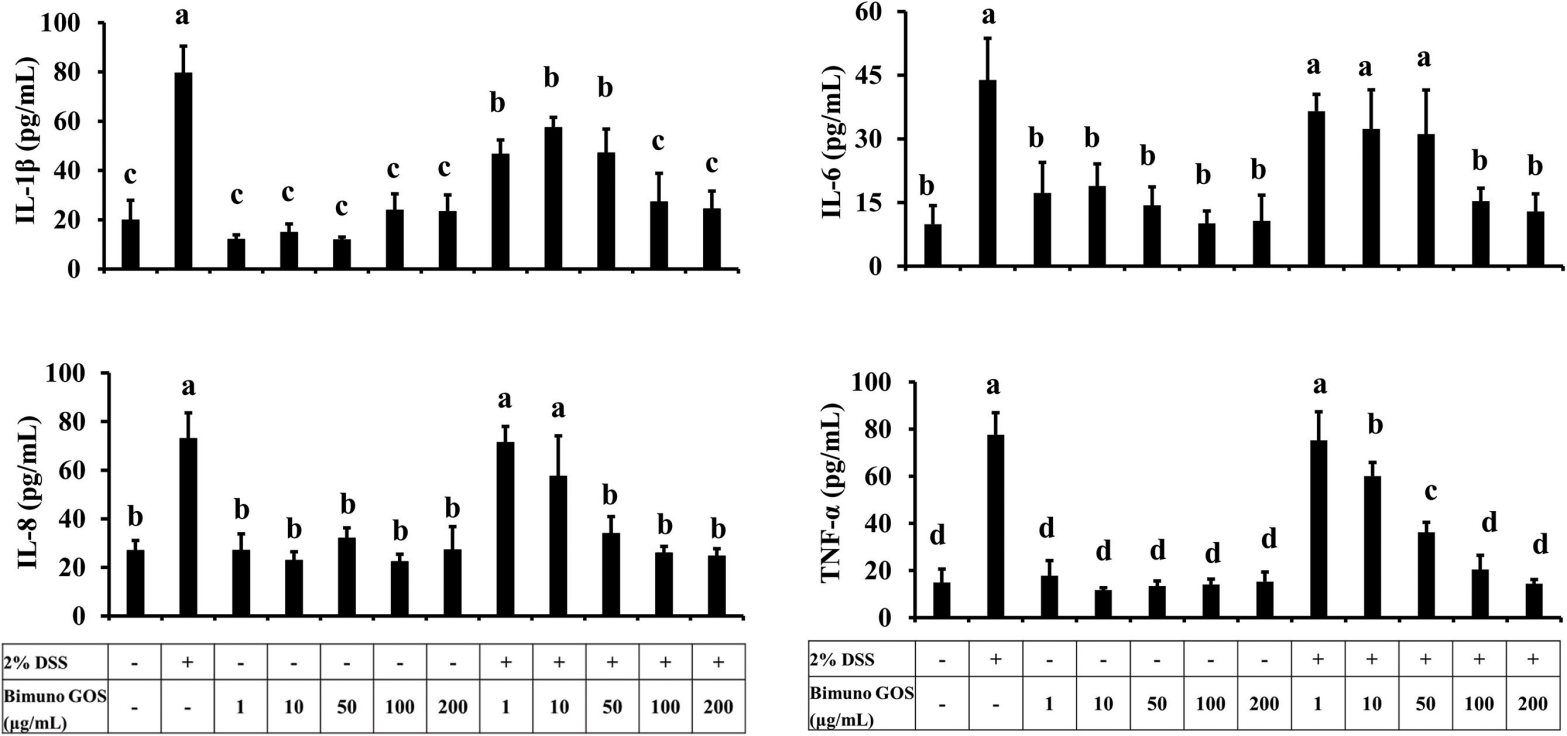
Pro-inflammatory cytokine secretion in presence of different concentrations of Bimuno GOS and/or DSS in Caco-2 cells. Cells were untreated, or treated with 2% DSS alone for 6 h, or treated with 100 or 200 μg/ml Bimuno GOS alone for 4 h, or treated with 2% DSS for 2 h and then with 100 or 200 μg/ml Bimuno GOS for further 4 h. Values represent means ± SD of three independent experiments, carried out in triplicate. Means without a common letter significantly differ, *P* < 0.05.

Considering the role of pro-inflammatory cytokines in the mucosal damages occurring in patients with UC, the principal cytokines involved in the UC pathogenesis were investigated in Caco-2 cells. In particular, secretion of IL-1β, IL-6, IL-8, and TNF-α was analyzed in Caco-2 cell culture supernatants after treatment with DSS alone, GOS alone, or first with DSS for 2 h and then GOS for the following 4 h. GOS alone did not induce apical secretion of IL-1β, IL-6, IL-8, and TNF-α by Caco-2 cells, while DSS induced a significant increase (*P* < 0.01) of all the pro-inflammatory cytokines, as compared to control untreated cells (Figure 5; Supplementary Tables S4a–d).

GOS at concentrations from 1 to 50 μg/ml administered 2 h after DSS did not change IL-1β secretion in comparison to DSS-treated cells, whereas 100 and 200 μg/ml GOS significantly reduced IL-1β secretion induced by DSS (*P* < 0.05) compared to DSS alone. However, this reduction in IL-1β secretion was still significantly higher compared to the control (*P* < 0.05, Supplementary Table S4a).

Similar results were obtained for IL-6, which was induced by DSS as compared to the control (*P* < 0.01), and was significantly reduced in cells first treated with DSS and then either with 100 or 200 μg/ml GOS (*P* < 0.01, Supplementary Table S4b).

DSS salt significantly increased IL-8 secretion, as compared to the control (*P* < 0.01). GOS concentrations ranging from 50 to 200 μg/ml were effective in reducing the IL-8 secretion in DSS-treated Caco-2 cells (*P* < 0.01), whereas GOS concentrations from 1 to 10 μg/ml were not able to counteract the DSS-induced IL-8 secretion (Supplementary Table S4c).

DSS salt also induced TNF-α secretion as compared to the control (*P* < 0.01), this increase was partially reduced by 50 μg/ml GOS (*P* < 0.05), and totally inhibited by treatment with 100 or 200 μg/ml GOS (*P* < 0.01, Supplementary Table S4d).

Overall, 100 and 200 μg/ml GOS concentrations were the most effective in reducing the pro-inflammatory cytokine secretion induced by DSS. In particular, we observed that treatment with 100 or 200 μg/ml GOS was able to reduce to control level IL-6, IL-8, and TNF-α (Figure 5).

### GOS Inhibited NF-kB Pathway Signaling Induced by DSS

**Figure 6:**
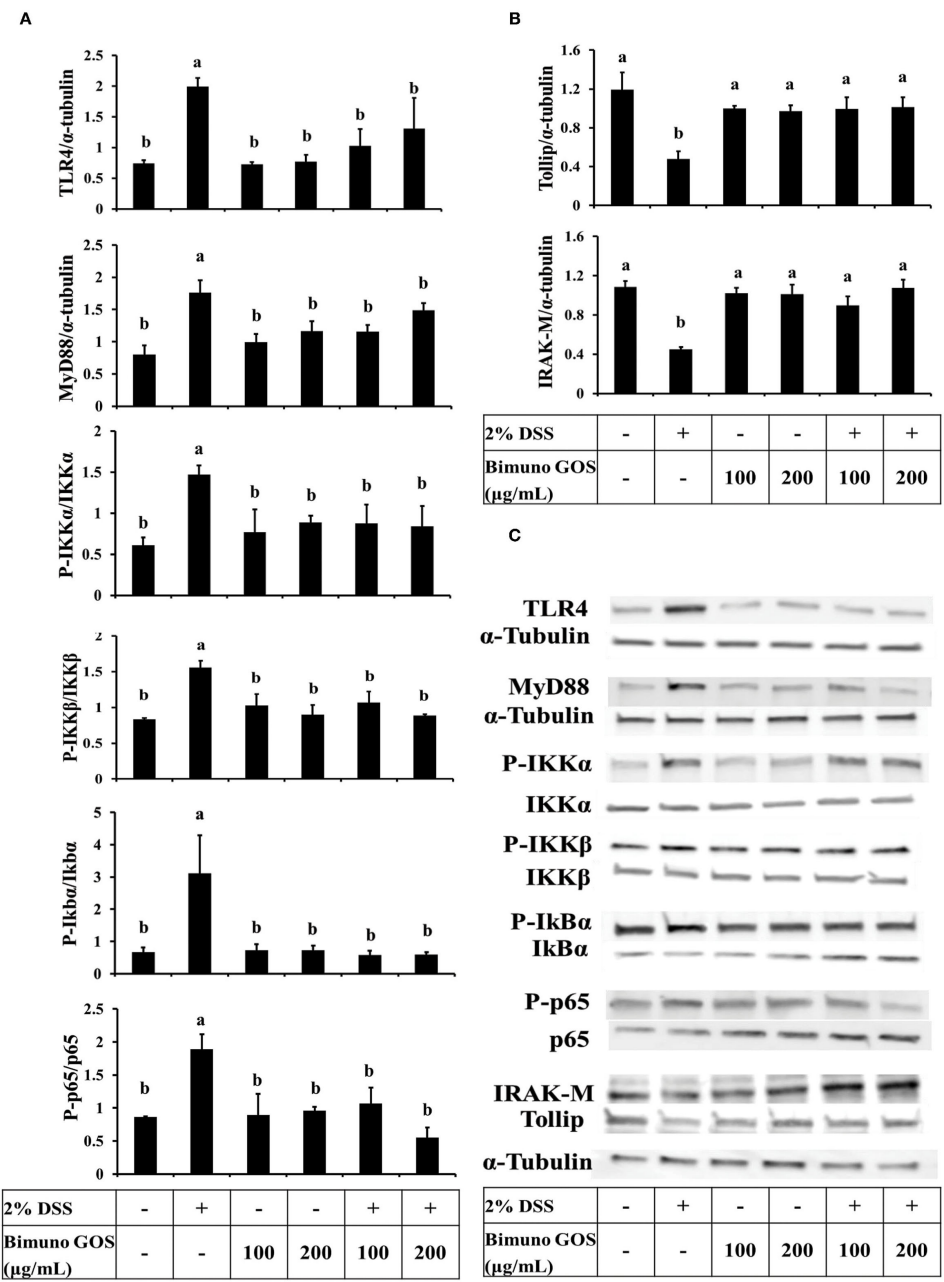
Inhibition of DSS induced-NF-kB signaling pathway by Bimuno GOS in Caco-2 cells. Cells were untreated, or treated with 2% DSS alone for 6 h, or treated with 2% DSS for 2 h and then with Bimuno GOS (100 or 200 μg/ml) for a further 4 h, or treated with 100 or 200 μg/ml Bimuno GOS alone for 4 h. Protein expression was analyzed by Western blot. The relative expression levels of TLR4, MyD88, Tollip, and IRAK-M were normalized to α-tubulin, whereas the phosphorylated IKK-α, IKK-β, IkB-α, and p65 were normalized to their corresponding unphosphorylated forms. **(A)** TLR4, MyD88, P-IKK-α, P-IKK-β, P-IkB-α, P-p65 (densitometric values). **(B)** Tollip and IRAK-M (densitometric values). **(C)** Representative Western blot of the analyzed proteins. Values represent means ± SD of three independent experiments, carried out in triplicate. Means without a common letter significantly differ, *P* < 0.05.

In order to clarify the mechanism of action of this specific GOS on the inflammatory cascade, the expression level of the key proteins involved in the activation of the NF-kB pathway was analyzed by Western blot. DSS induced a significant increase of expression of TLR4, MyD88, P-IKKα, P-IKKβ (*P* < 0.05), P-IkBα, and P-p65 (*P* < 0.01), as compared to the control (Figure 6A; Supplementary Tables S5a–f). In addition, the expression of Tollip and IRAK-M, negative regulators of NF-kB cascade, was significantly reduced (*P* < 0.01) as compared to the control (Figure 6B; Supplementary Tables S5g,h). No significant differences for all the analyzed proteins could be observed in cells treated with 100 or 200 μg/ml GOS alone, as compared to the control.

On the other hand, Caco-2 cells treated first with DSS and then with GOS had similar expression levels of all the activator proteins involved in the NF-kB inflammatory pathway (Figure 6A) and of Tollip and IRAK-M negative regulators (Figures 6B,C), as compared to control cells.

**Figure 7:**
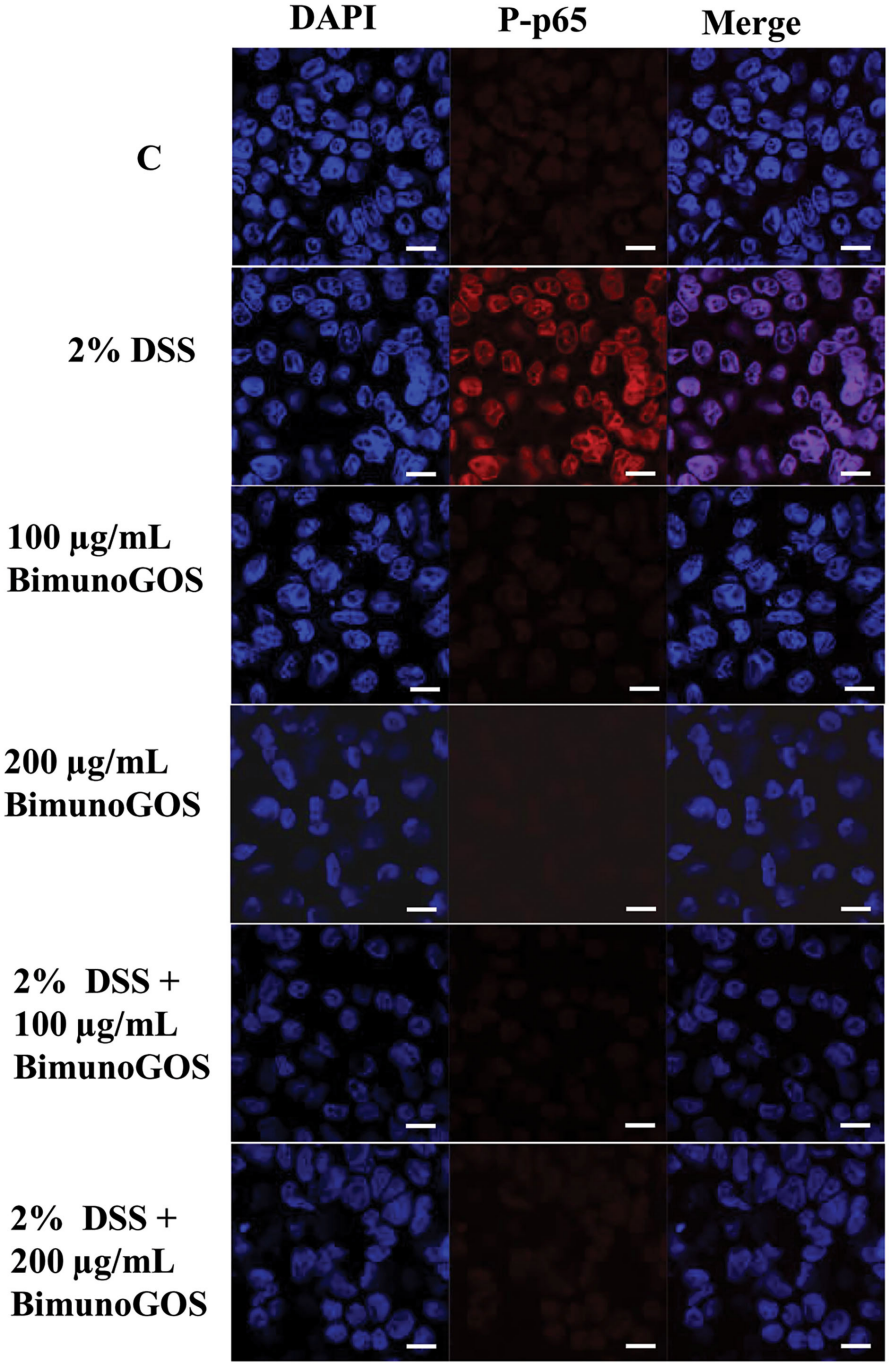
Inhibition of DSS induced-P-p65 translocation by Bimuno GOS in Caco-2 cells. Cells were untreated (C), or treated with 2% DSS for 6 h, or treated with Bimuno GOS (100 or 200 μg/ml) for 4 h, or treated with 2% DSS for 2 h and then with Bimuno GOS (100 or 200 μg/ml) for further 4 h. Cell nuclei were stained with DAPI, while P-p65 was labeled with rabbit polyclonal anti-P-p65 antibody, followed by TRITC-conjugated secondary antibody. Each figure is representative of three independent assays (63 × magnification). Bars represent 10 μm.

Data on P-p65 protein expression levels were confirmed by immunolocalization analysis. Immunolabeling of P-p65 showed that treatment with DSS induced a strong migration into the nucleus of P-p65, as indicated by the high fluorescence signal intensity, whereas in control cells or in cells treated with 100 or 200 μg/ml GOS alone the positive signal into the nucleus was slight or absent. In agreement with the results of Western blot analysis, treatment with 100 or 200 μg/ml GOS was able to inhibit the P-p65 migration induced by DSS treatment (Figure 7).

All these results indicate that GOS was able to counteract the DSS-induced inflammatory NF-kB cascade activation.

### Maintenance of Tight and Adherent Junction Protein Localization by GOS

**Figure 8:**
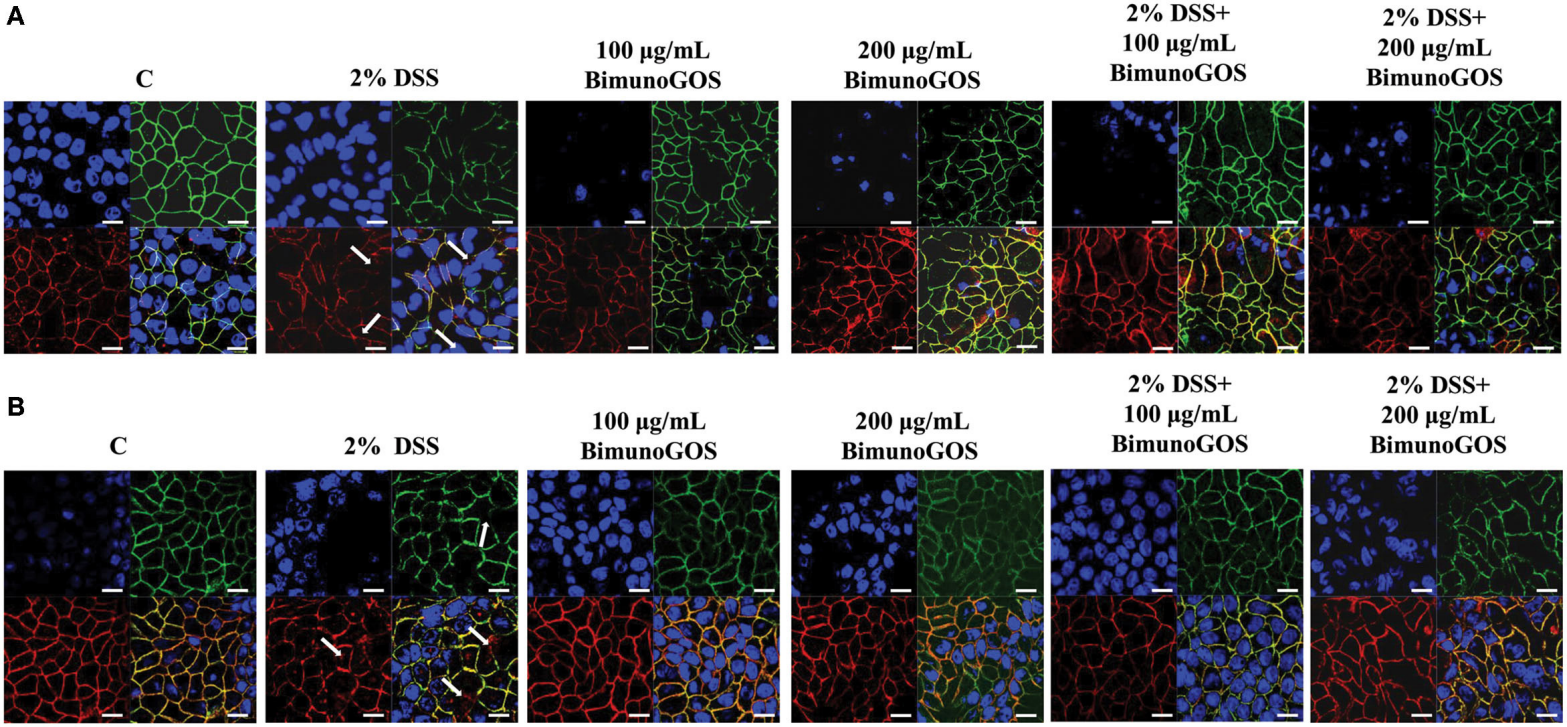
Localization of tight and adherent junction proteins in Caco-2 cells. Cells were either untreated (C), or treated with 2% DSS for 6 h, or treated with Bimuno GOS (100 or 200 μg/ml) for 4 h, or treated with 2% DSS for 2 h and then with Bimuno GOS (100 or 200 μg/ml) for further 4 h. Cells were labeled with specific primary antibodies for tight **(A)** and adherent **(B)** junction proteins, followed by TRITC- and FITC-conjugated secondary antibodies for occludin and ZO-1, and for β-catenin and E-cadherin, respectively. Cell nuclei were stained with DAPI. Dissociation of occludin from the membrane is indicated by arrows. Each figure is representative of three independent immunofluorescence assays (63 × magnification). Bars represent 10 μm.

In order to evaluate the ability of the GOS to counteract the membrane barrier damage induced by DSS in Caco-2 cells, immunolocalization of the principal tight and adherent junction proteins was performed. As reported in Figure 8A, DSS induced several damages in both ZO-1 and occludin protein distribution, as ZO-1 resulted in discontinuous cell boundaries, whereas occludin disappeared from cell boundaries and partly localized in the cell cytoplasm. Several damages induced by DSS were also present in the adherens junctions. Indeed, DSS induced the disappearance of both E-cadherin and β-catenin from the cell boundaries associated with a loss of co-localization of the two proteins. A total of 100 and 200 μg/ml GOS were able to rescue the damages induced by DSS in both tight and adherent junction protein distribution, as shown in Figure 8B.

## Discussion

In this study, the potential benefits of a specific GOS (Bimuno GOS) for UC were tested in an established cell culture model of gut inflammation, i.e., the Caco-2 intestinal cell line, where DSS was used to induce gut inflammation and cell damage.

This study demonstrates that the specific GOS tested can counteract UC-like inflammatory characteristics, gut permeability markers, and tissue damages induced by DSS in the *in vitro* model. Several *in vivo* and *in vitro* studies have shown that in UC cellular junctions damages are associated with increased permeability of intestinal mucosa (44, 45). In line with these studies, we observed that DSS treatment induced cell junction opening, increased monolayer permeability, and the disappearance of tight and adherent junction proteins. Importantly, we observed that GOS on its own did not affect membrane permeability, as indicated by TEER and Papp measurements, which is essential for being a safe and potentially effective way of improving the gut inflammatory state in IBD. All damages induced by DSS to the epithelial barrier were reversed after treatment with GOS, including the DSS-induced cell damage and permeability determined by the localization of the tight junction proteins ZO-1 and occludin, and the adherent junction proteins E-cadherin and β-catenin. This demonstrates that the specific GOS could be beneficial in preventing gut barrier disruption in challenging conditions such as gut inflammation.

In active UC, expression of pro-inflammatory cytokines such as IL-1β, IL-6, IL-8, and TNF-α is usually increased, and pro-inflammatory cytokines have been clearly demonstrated to play a key role in epithelial junction remodeling and disruption (46, 47). In the present study, we demonstrated that GOS on its own did not induce the secretion of pro-inflammatory cytokines IL-1β, IL-6, IL-8, and TNF-α, whereas DSS alone as the pro-inflammatory stimulant triggered secretion of all these cytokines through the activation of the NF-kB pathway, as indicated by translocation of the phosphorylated form of p65 into the nucleus. The secretion of IL-1β, IL-6, IL-8, and TNF-α was significantly decreased when GOS was added to DSS-treated cells, compared to the DSS treatment alone. Our results are in agreement with those obtained by Hwang and colleagues, who observed that inflammatory status induced in Caco-2 cells by LPS challenge was reduced by treatment with low-molecular-weight polysaccharides showing prebiotic activity (48). It was also shown that low-molecular-weight fucoidan and high-stability fucoxanthin were able to inhibit IL-1β and TNF-α secretion to promote IL-10 and IFN-γ secretion in cells treated with LPS, indicating that these compounds could exert anti-inflammatory activity on intestinal cells. In this study, the most effective GOS concentrations in dampening pro-inflammatory cytokine secretion were 100 and 200 μg/ml. A more detailed analysis demonstrated that proteins of the NF-kB signaling pathway were involved in the pro-inflammatory cascade induced by DSS. Indeed, 2% DSS induced the NF-kB inflammatory pathway, as shown by the increase of TLR4, MyD88, P-IKKα, P-IKKβ, P-IkBα, and P-p65 and the reduction of negative regulators Tollip and IRAK-M protein expression levels. Bimuno GOS inhibited the DSS-induced expression levels of TLR4, MyD88, P-IKKα, P-IKKβ, P-IkBα, and P-p65, which were restored to control levels.

Our results are in agreement with those shown in a previous study conducted by Wu et al., where Caco-2 cells were treated with several pro-inflammatory agents, however, other prebiotics, such as inulin or FOS, were used (38). Interestingly, we also observed that GOS was able to restore the expression levels of NF-kB negative regulators, Tollip and IRAK-M. Although the role of Tollip and IRAK-M in IBD is not yet clarified, in a study conducted on biopsies from CD and UC patients, Fernandes et al. reported a reduced Tollip expression, similar to our findings (49). However, in contrast to our data reported here, these authors observed an increase in IRAK-M, and concluded that the up-regulation of IRAK-M was a mechanism to counteract the high level of inflammation. Nevertheless, the role of IRAK-M has been highlighted in the IRAK-M ^−/−^ mice model orally treated with DSS (50). In line with our findings, the authors showed that IRAK-M plays a key role in downregulating the induction and progression of DSS colitis through the modulation of proinflammatory cytokines such as TNF-α and IL-6. The role of IRAK-M has been extensively investigated in several studies reporting its role in LPS and DSS inflammatory state induction. Both LPS and DSS act through TLR4 signaling and modulate NF-kB and MAPK cascade in *in vitro* and *in vivo* studies. IRAK-M deficiency was shown to be responsible for the intestinal inflammation onset, suggesting a possible impairment in the negative regulation of TLR signaling causing IBD (51, 52). In our model, the ability of GOS to modulate the expression of both negative regulators Tollip and IRAK-M is important, since they were able to block the pro-inflammatory NF-kB pathway by inhibiting the transcription of inflammatory mediators.

The role of pro-inflammatory cytokines such as IL-1β, IL-6, IL-8, and TNF-α in UC pathogenesis has been well described in the literature. These cytokines are involved in pro-inflammatory responses through immune cells recruitment, followed by amplification and propagation of inflammation (53). In our model, Caco-2 cells exposed to DSS showed increased secretion of all the analyzed pro-inflammatory cytokines, as compared to the control. GOS was able to abolish the increase of pro-inflammatory cytokines induced by DSS and this indicates that GOS is able to reduce inflammation. The involvement of cytokines in the epithelial damages of UC is well known, however, only a few studies have analyzed the effect of prebiotic molecules on inflammatory process repair, as well as their ability to modulate cellular response without involving microbiota (38, 48, 54). In all these studies, the authors showed that prebiotics was able to induce an immunomodulatory effect by modulating the NF-kB pathway and cytokine secretion. Pistol and colleagues reported that in LPS-pretreated Caco-2 cells a synbiotic (grape extract plus lactobacilli mixture) treatment for 24 h induced a decrease of inflammatory cytokine secretion associated with the prevention of MAPK and NF-kB markers induction. In addition, Wu and colleagues (38) showed in an *in vitro* model of Caco-2 cells treated with FOS, prebiotics could directly influence the signal transduction mediated by protein kinases.

Prebiotics have been also tested in the context of irritable bowel syndrome (IBS). In an *in vitro* model of IBS, obtained by infection of Caco-2 cells with *Salmonella typhimurium* and post-infection treatment with a prebiotic blend (FOS plus GOS), prebiotics were shown to inhibit the pro-inflammatory cytokine secretion by suppressing inflammation, and this activity was not mediated by microbiota (55). Similarly, in our study, we show that GOS was able to reduce the inflammatory status in an *in vitro* UC-like model by acting in a microbiota-independent way. From all our data we can speculate that in DSS treated cells, GOS was able to counteract the inflammatory status and the membrane barrier damage induced by inflammatory cytokines that reverted to control level. The ability of GOS to regulate immune response was previously suggested, as it has been shown to be effective in modulating cytokine secretion in intestinal cells through TLR4 binding (56), as well as through a direct effect on intestinal cell transcriptome by modulating the expression of several genes, including some involved in antimicrobial activity and inflammatory response (36). According to previous studies, our results strongly suggest that a specific prebiotic can have a direct effect on the regulation of inflammation.

Overall, the present study identifies mechanisms of how GOS can support gut cells by improving their function, including gut barrier function, and decreasing inflammation in the context of UC, through modulation of the NF-kB pathway and pro-inflammatory cytokine secretion. The current study, although with the limitations of being a preclinical experimental setup, shows the potential of the specific GOS for the management of a challenging condition such as IBD, where gut barrier integrity and function are compromised by chronic inflammation.

We used differentiated Caco-2 epithelial cells as a suitable, reliable, and widely used model of intestinal barrier, that mimics the *in vivo* intestinal mucosa and allows the understanding of some mechanisms of action. We demonstrated that Bimuno GOS has direct effects in such model, by reducing pro-inflammatory cytokines and damage caused by DSS. It is noteworthy that in addition to direct effects, the Bimuno GOS used in this study has been demonstrated to have potent indirect effects involving growth stimulation of beneficial gut bacteria such as bifidobacteria (26, 33) on gut microbiota modulation, which in turn have been associated with immune system modulation (57). The gut-immune interplay is pivotal in the induction and maintenance of a non-inflammatory status and a local tolerogenic environment (58, 59). A recent study in a cohort of patients with UC demonstrated that the administration of Bimuno GOS for 6 weeks results in overall normalization of stools and reduced incidence and severity of loose stools, in addition to decreased urgency (26). A subset of patients in the remission stage had increased bifidobacterial counts. Although Wilson et al. study (26) did not investigate immune markers in patients with UC, it demonstrated a clinical improvement. The results from the current study, using the DSS model suggest a potential mechanism of action for the observed clinical outcomes in Wilson et al. (26). It remains to be explored how the immune markers are affected in the cohort of UC patients taking a prebiotic.

Finally, it is common knowledge that GOS is a mixture of multiple oligosaccharide structures. The specific GOS in this study had a relatively high abundance of the lower DP fractions, which is inherent to the manufacturing of GOS by enzymatic processes. Interestingly, a previous study conducted by Newburg and colleagues (60) has shown that trisaccharide (DP3) structures in particular and at a relatively high quantity in this GOS have strong immunological, i.e., anti-inflammatory responses in different human intestinal cell lines. This warrants further research into the specific structure—bioactivity relationships of individual GOS structures to further understand their contribution to biological, and thus immune activities. More detailed GOS structure analyses and comparisons between different GOS's, including Bimuno GOS used in the present study, have previously been published by Hernandez-Hernandez et al. (61) and Van Leeuwen et al. (62). Further to that, it should also be emphasized that generic extrapolation of effects between different GOS's cannot be made. Structure analyses have shown clear differences between individual GOS types (61, 62), and therefore each GOS product or GOS-derived fraction should be tested separately for their biological properties and activities in future research.

## Conclusions

In conclusion, using DSS-treated Caco-2 cells as an *in vitro* UC model, we suggest that the specific GOS tested in this study can be a safe and effective way not only to modulate gut inflammation but also to potentially prevent or restore gut barrier disruption and further improve efficacy in inducing remission, although *in vivo* validation is necessary. Currently, various pharmaceutical options are available for the treatment of IBD, however, all with their own limitations related to efficacy, side effects, and costs (10, 63). A prebiotic supplement such as the specific GOS tested in this study could be an attractive therapeutic agent or an add-on to other treatment options including enteral nutrition for managing IBD.

## Data Availability Statement

The raw data supporting the conclusions of this article will be made available by the authors, without undue reservation.

## Author Contributions

AF and RG: conceptualization, methodology, and validation. AF and MR: investigation, formal analysis, visualization, writing—original draft, supervision, project administration, and funding acquisition. AM and LH: writing—review and editing. All authors have read and approved the final version of the manuscript and participated to the decision to submit the manuscript for publication.

## Funding

The authors declare that this study received part of the funding from Clasado Biosciences Ltd.

## Conflict of Interest

AM and LH are currently employees of Clasado Biosciences. RG was an employee of Clasado Biosciences when the work was performed. AM, LH, and RG had no access to raw data and they did not participate in data analysis. The authors declare that this study received funding from Clasado Biosciences. The funder had the following involvement with the study: Clasado co-founded the study but was not involved in the data collection and analysis, as well as interpretation of data. Clasado indeed participated to writing and revision of the present manuscript and agreed to submit it for publication. The remaining authors declare that the research was conducted in the absence of any commercial or financial relationships that could be construed as a potential conflict of interest.

## Publisher's Note

All claims expressed in this article are solely those of the authors and do not necessarily represent those of their affiliated organizations, or those of the publisher, the editors and the reviewers. Any product that may be evaluated in this article, or claim that may be made by its manufacturer, is not guaranteed or endorsed by the publisher.

## References

[1] GBD 2017. Inflammatory Bowel Disease Collaborators. The global, regional, and national burden of inflammatory bowel disease in 195 countries and territories, 1990-2017: a systematic analysis for the Global Burden of Disease Study 2017. Lancet Gastroenterol Hepatol. (2020) 5:17–30. 10.1016/S2468-1253(19)30333-4 31648971 PMC7026709

[2] Gasparini RG Sassaki LY Saad-Hossne R. Inflammatory bowel disease epidemiology in São Paulo State, Brazil. Clin Exp Gastroenterol. (2018) 11:423–9. 10.2147/CEG.S176583 30464570 PMC6214600

[3] Roselli M Finamore A. Use of synbiotics for ulcerative colitis treatment. Curr Clin Pharmacol. (2020) 15:174–82. 10.2174/1574884715666191226120322 31878863

[4] Kobayashi T Siegmund B Le Berre C Wei SC Ferrante M Shen B. Ulcerative colitis. Nat Rev Dis Primers. (2020) 6:74. 10.1038/s41572-020-0205-x 32913180

[5] Finamore A Peluso I Cauli O. Salivary stress/immunological markers in crohn's disease and ulcerative colitis. Int J Mol Sci. (2020) 21:8562. 10.3390/ijms21228562 33202858 PMC7698267

[6] Schulzke JD Ploeger S Amasheh M Fromm A Zeissig S Troeger H. Epithelial tight junctions in intestinal inflammation. Ann N Y Acad Sci. (2009) 1165:294–300. 10.1111/j.1749-6632.2009.04062.x 19538319

[7] Szebeni B Veres G Dezsõfi A Rusai K Vannay A Mraz M. Increased expression of Toll-like receptor (TLR) 2 and TLR4 in the colonic mucosa of children with inflammatory bowel disease. Clin Exp Immunol. (2008) 151:34–41. 10.1111/j.1365-2249.2007.03531.x 17991289 PMC2276924

[8] Steinbach EC Plevy SE. The role of macrophages and dendritic cells in the initiation of inflammation in IBD. Inflamm Bowel Dis. (2014) 20:166–75. 10.1097/MIB.0b013e3182a69dca 23974993 PMC4098861

[9] Candia E Díaz-Jiménez D Langjahr P Núñez LE. de la Fuente M Farfán N. Increased production of soluble TLR2 by lamina propria mononuclear cells from ulcerative colitis patients. Immunobiology. (2012) 217:634–42. 10.1016/j.imbio.2011.10.023 22101184

[10] Mowat C Cole A Windsor A Ahmad T Arnott I Driscoll R. IBD Section of the british society of gastroenterology. Guidelines for the management of inflammatory bowel dis-ease in adults. Gut. (2011) 60:571–607. 10.1136/gut.2010.224154 21464096

[11] Guo XY Liu XJ Hao JY. Gut microbiota in ulcerative colitis: insights on pathogenesis and treatment. J Dig Dis. (2020) 21:147–59. 10.1111/1751-2980.12849 32040250

[12] Pei LY Ke YS Zhao HH Wang L Jia C Liu WZ. Role of colonic microbiota in the pathogenesis of ulcerative colitis. BMC Gastroenterol. (2019) 19:10. 10.1186/s12876-019-0930-3 30642266 PMC6332670

[13] Alam MT Amos GCA Murphy ARJ Murch S Wellington EMH Arasaradnam RP. Microbial imbalance in inflammatory bowel disease patients at different taxonomic levels. Gut Pathog. (2020) 12:1. 10.1186/s13099-019-0341-6 31911822 PMC6942256

[14] Manichanh C Borruel N Casellas F Guarner F. The gut microbiota in IBD. Nat Rev Gastroenterol Hepatol. (2012) 9:599–608. 10.1038/nrgastro.2012.152 22907164

[15] Li YH Adam R Colombel JF Bian ZX. A characterization of pro-inflammatory cytokines in dextran sulfate sodium-induced chronic relapsing colitis mice model. Int Immunopharmacol. (2018) 60:194–201. 10.1016/j.intimp.2018.05.001 29747125

[16] Toutounji M Wanes D El-Harakeh M El-Sabban M Rizk S Naim HY. Dextran sodium sulfate-induced impairment of protein trafficking and alterations in membrane composition in intestinal Caco-2 cell line. Int J Mol Sci. (2020) 21:2726. 10.3390/ijms21082726 32326391 PMC7215722

[17] Li Q Liang X Guo N Hu L Prasad EM Wu Y. Protective effects of Bee pollen extract on the Caco-2 intestinal barrier dysfunctions induced by dextran sulfate sodium. Biomed Pharmacother. (2019) 117:109200. 10.1016/j.biopha.2019.109200 31387194

[18] Araki Y Sugihara H Hattori T. In vitro effects of dextran sulfate sodium on a Caco-2 cell line and plausible mechanisms for dextran sulfate sodium-induced colitis. Oncol Rep. (2006) 16:1357–62. 10.3892/or.16.6.1357 17089061

[19] Kang EA Choi HI Hong SW Kang S Jegal HY Choi EW. Extracellular vesicles derived from kefir grain lactobacillus ameliorate intestinal inflammation via regulation of proinflammatory pathway and tight junction integrity. Biomedicines. (2020) 8:522. 10.3390/biomedicines8110522 33233771 PMC7709018

[20] Gibson GR Hutkins R Sanders ME Prescott SL Reimer RA Salminen SJ. Expert consensus document: the international scientific association for probiotics and prebiotics (ISAPP) consensus statement on the definition and scope of prebiotics. Nat Rev Gastroenterol Hepatol. (2017) 14:491–502. 10.1038/nrgastro.2017.75 28611480

[21] Gibson GR Probert HM Van Loo J Rasrall RA Roberfroid MB. Dietary modulation of the human colonic microbiota: introducing the concept of prebiotics. Nutr Res Rev. (2004) 17:259–75. 10.1093/jn/125.6.1401 19079930

[22] Roberfroid M. Dietary fiber, inulin, and oligofructose: a review comparing their physiological effects. Crit Rev Food Sci Nutr. (1993) 33:103–48. 10.1080/10408399309527616 8257475

[23] Novak M Vetvicka V. Beta-glucans, history, and the present: immunomodulatory aspects and mechanisms of action. J Immunotoxicol. (2008) 5:47–57. 10.1080/15476910802019045 18382858

[24] Niittynen L Kajander K Korpela R. Galacto-oligosaccharides and bowel function. Scand J Food Nutr. (2007) 51:62–6. 10.1080/17482970701414596

[25] Davani-Davari D Negahdaripour M Karimzadeh I Seifan M Mohkam M Masoumi SJ. Prebiotics: Definition, Types, Sources, Mechanisms, and Clinical Applications. Foods. (2019) 8:92. 10.3390/foods8030092 30857316 PMC6463098

[26] Wilson B Eyice Ö Koumoutsos I Lomer MC Irving PM Lindsay JO. Prebiotic galactooligosaccharide supplementation in adults with ulcerative colitis: exploring the impact on peripheral blood gene expression, gut microbiota, and clinical symptoms. Nutrients. (2021) 13:3598. 10.3390/nu13103598 34684597 PMC8537576

[27] Guarino MPL Altomare A Emerenziani S Di Rosa C Ribolsi M Balestrieri P. Mechanisms of action of prebiotics and their effects on gastro-intestinal disorders in adults. Nutrients. (2020) 12:1037. 10.3390/nu12041037 32283802 PMC7231265

[28] Akram W Garud N Joshi R. Role of inulin as prebiotics on inflammatory bowel disease. Drug Discov Ther. (2019) 13:1–8. 10.5582/ddt.2019.01000 30880316

[29] Parada Venegas D. De la Fuente MK Landskron G González MJ Quera R Dijkstra G. Short Chain Fatty Acids (SCFAs)-mediated gut epithelial and immune regulation and its relevance for inflammatory bowel diseases. Front Immunol. (2019) 10:277. 10.3389/fimmu.2019.00277 30915065 PMC6421268

[30] Casellas F Borruel N Torrejon A Varela E Antolin M Guarner F. Oral oligofructose-enriched inulin supplementation in acute ulcerative colitis is well tolerated and associated with lowered faecal calprotectin. Aliment Pharmacol Ther. (2007) 25:1061–7. 10.1111/j.1365-2036.2007.03288.x 17439507

[31] Brummer Y Kaviani M Tosh SM. Structural and functional characteristics of dietary fibre in beans, lentils, peas and chickpeas. Food Res Int. (2015) 67:117–25. 10.1016/j.foodres.2014.11.009

[32] Vulevic J Rastall RA Gibson GR. Developing a quantitative approach for determining the in vitro prebiotic potential of dietary oligosaccharides. FEMS Microbiol Lett. (2004) 236:153–9. 10.1016/j.femsle.2004.05.036 15212805

[33] Depeint F Tzortzis G Vulevic J I'anson K Gibson GR. Prebiotic evaluation of a novel galactooligosaccharide mixture produced by the enzymatic activity of *Bifidobacterium bifidum* NCIMB 41171, in healthy humans: a randomized, double-blind, crossover, placebo-controlled intervention study. Am J Clin Nutr. (2008) 87:785–91. 10.1093/ajcn/87.3.785 18326619

[34] Vulevic J Juric A Walton GE Claus SP Tzortzis G Toward RE. Influence of galacto-oligosaccharide mixture (B-GOS) on gut microbiota, immune parameters and metabonomics in elderly persons. Br J Nutr. (2015) 114:586–95. 10.1017/S0007114515001889 26218845

[35] Perdijk O van Baarlen P Fernandez-Gutierrez MM van den Brink E Schuren FHJ Brugman S. Sialyllactose and galactooligosaccharides promote epithelial barrier functioning and distinctly modulate micro-biota composition and short chain fatty acid production in vitro. Front Immunol. (2019) 10:94. 10.3389/fimmu.2019.00094 30809221 PMC6380229

[36] Lafontaine GMF Fish NM Connerton IF. In vitro evaluation of the effects of commercial prebiotic GOS and FOS products on human colonic Caco-2 cells. Nutrients. (2020) 12:1281. 10.3390/nu12051281 32366023 PMC7282019

[37] Lehmann S Hiller J van Bergenhenegouwen J Knippels LM Garssen J Traidl-Hoffmann C. In vitro evidence for immune-modulatory properties of non-digestible oligosaccharides: direct effect on human monocyte derived dendritic cells. PLoS ONE. (2015) 10:e0132304. 10.1371/journal.pone.0132304 26148091 PMC4493044

[38] Wu RY Määttänen P Napper S Scruten E Li B Koike Y. Non-digestible oligosaccharides directly regulate host kinome to modulate host inflammatory responses without alterations in the gut microbiota. Microbiome. (2017) 5:135. 10.1186/s40168-017-0357-4 29017607 PMC5635512

[39] Del Fabbro S Calder PC Childs CE. Microbiota-independent immunological effects of non-digestible oligosaccharides in the context of inflammatory bowel diseases. Proc Nutr Soc. (2020) 2020:1–11. 10.1017/S0029665120006953 32345388

[40] Sambuy Y De Angelis I Ranaldi G Scarino ML Stammati A Zucco F. The Caco-2 cell line as a model of the intestinal barrier: influence of cell and culture-related factors on Caco-2 cell functional characteristics. Cell Biol Toxicol. (2005) 21:1–26. 10.1007/s10565-005-0085-6 15868485

[41] Ferruzza S Sambuy Y Onetti-Muda A Nobili F Scarino ML. Copper toxicity to tight junctions in the human intestinal Caco-2 cell line BT. In: Massaro EJ editor. Handbook of Copper Pharmacology and Toxicology. Totowa, NJ: Humana Press (2002). p. 397–416

[42] Hubatsch I Ragnarsson EG Artursson P. Determination of drug permeability and prediction of drug absorption in Caco-2 monolayers. Nat Protoc. (2007) 2:2111–9. 10.1038/nprot.2007.303 17853866

[43] Finamore A Roselli M Imbinto A Seeboth J Oswald IP Mengheri E. Lactobacillus amylovorus inhibits the TLR4 inflammatory signaling triggered by enterotoxigenic Escherichia coli via modulation of the negative regulators and involvement of TLR2 in intestinal Caco-2 cells and pig explants. PLoS ONE. (2014) 9:e94891. 10.1371/journal.pone.0094891 24733511 PMC3986366

[44] Lechuga S Ivanov AI. Disruption of the epithelial barrier during intestinal inflammation: quest for new molecules and mechanisms. Biochim Biophys Acta Mol Cell Res. (2017) 1864:1183–94. 10.1016/j.bbamcr.2017.03.007 28322932 PMC5507344

[45] Kucharzik T Walsh SV Chen J Parkos CA Nusrat A. Neutrophil transmigration in inflammatory bowel disease is associated with differential expression of epithelial inter-cellular junction proteins. Am J Pathol. (2001) 159:2001–9. 10.1016/S0002-9440(10)63051-9 11733350 PMC1850599

[46] Leppkes M Neurath MF. Cytokines in inflammatory bowel diseases - Update 2020. Pharmaco Res. (2020) 158:104835. 10.1016/j.phrs.2020.104835 32416212

[47] Luissint AC Parkos CA Nusrat A. Inflammation and the intestinal barrier: leukocyte-epithelial cell interactions, cell junction remodeling, and mucosal repair. Gastroenterology. (2016) 151:616–32. 10.1053/j.gastro.2016.07.008 27436072 PMC5317033

[48] Hwang PA Phan NN Lu WJ Ngoc Hieu BT Lin YC. Low-molecular-weight fucoidan and high-stability fucoxanthin from brown seaweed exert prebiotics and an-ti-inflammatory activities in Caco-2 cells. Food Nutr Res. (2016) 60:32033. 10.3402/fnr.v60.32033 27487850 PMC4973444

[49] Fernandes P MacSharry J Darby T Fanning A Shanahan F Houston A. Differential expression of key regulators of Toll-like receptors in ulcerative colitis and Crohn's disease: a role for Tollip and peroxisome proliferator-activated receptor gamma? Clin Exp Immunol. (2016) 183:358–68. 10.1111/cei.12732 26462859 PMC4750602

[50] Berglund M Melgar S Kobayashi KS Flavell RA Hörnquist EH Hultgren OH. IL-1 receptor-associated kinase M downregulates DSS-induced colitis. Inflamm Bowel Dis. (2010) 16:1778–51. 10.1002/ibd.21287 20848470

[51] Hubbard LL Moore BB. IRAK-M regulation and function in host defense and immune homeostasis. Infect Dis Rep. (2010) 2:e9. 10.4081/idr.2010.e9 21390243 PMC3049547

[52] Ren G Sun A Deng C Zhang J Wu X Wei X. The anti-inflammatory effect and potential mechanism of cardamonin in DSS-induced colitis. Am J Physiol Gastrointest Liver Physiol. (2015) 309:G517–27. 10.1152/ajpgi.00133.2015 26251468 PMC4593824

[53] Sanchez-Munoz F Dominguez-Lopez A Yamamoto-Furusho JK. Role of cytokines in inflammatory bowel disease. World J Gastroenterol. (2008) 14:4280–8. 10.3748/wjg.14.4280 18666314 PMC2731177

[54] Pistol GC Marin DE Dragomir C Taranu I. Synbiotic combination of prebiotic grape pomace extract and probiotic Lactobacillus sp. reduced important intestinal inflammatory markers and in-depth signalling mediators in lipopolysaccharide-treated Caco-2 cells. Br J Nutr. (2019) 121:291–305. 10.1017/S0007114518003410 30565527

[55] Chen Q Ren Y Lu J Bartlett M Chen L Zhang Y. A novel prebiotic blend product prevents irritable bowel syndrome in mice by improving gut microbiota and modulating immune response. Nutrients. (2017) 9:1341. 10.3390/nu9121341 29232851 PMC5748791

[56] Ortega-González M Ocón B Romero-Calvo I Anzola A Guadix E Zarzuelo A. Nondigestible oligosaccharides exert nonprebiotic effects on intestinal epithelial cells enhancing the immune response via activation of TLR4-NFκB. Mol Nutr Food Res. (2014) 58:384–93. 10.1002/mnfr.201300296 24039030

[57] Vulevic J Drakoularakou A Yaqoob P Tzortzis G Gibson GR. Modulation of the fecal microflora profile and immune function by a novel trans-galactooligosaccharide mixture (B-GOS) in healthy elderly volunteers. Am J Clin Nutr. (2008) 88:1438–46. 10.3945/ajcn.2008.26242 18996881

[58] Geuking MB Cahenzli J Lawson MA Ng DC Slack E Hapfelmeier S. Intestinal bacterial colonization induces mutualistic regulatory T cell responses. Immunity. (2011) 34:794–806. 10.1016/j.immuni.2011.03.021 21596591

[59] Zoumpopoulou G Tsakalidou E Dewulf J Pot B Grangette C. Differential crosstalk between epithelial cells, dendritic cells and bacteria in a co-culture model. Int J Food Microbiol. (2009) 131:40–51. 10.1016/j.ijfoodmicro.2008.12.037 19264370

[60] Newburg DS Ko JS Leone S Nanthakumar NN. Human milk oligosaccharides and synthetic galactosyloligosaccharides contain 3′-, 4′-, and 6′-Galactosyllactose and attenuate inflammation in human T84, NCM-460, and H4 cells and intestinal tissue ex vivo. J Nutr. (2016) 146:358–67. 10.3945/jn.115.220749 26701795 PMC4725434

[61] Hernández-Hernández O Calvillo I Lebrón-Aguilar R Moreno FJ Sanz ML. Hydrophilic interaction liquid chromatography coupled to mass spectrometry for the characterization of prebiotic galactooligosaccharides. J Chromatogr A. (2012) 1220:57–67. 10.1016/j.chroma.2011.11.047 22189297

[62] Van Leeuwen SS Kuipers BJH Dijkhuizen L Kamerling JP. Comparative structural characterization of 7 commercial galacto-oligosaccharide (GOS) products. Carbohydrate Res. (2016) 425:48–58. 10.1016/j.carres.2016.03.006 27035911

[63] Dignass A Eliakim R Magro F Maaser C Chowers Y Geboes K. Crohns Colitis. Second European evidence-based consensus on the diagnosis and management of ulcerative colitis part 1: definitions and diagnosis. Crohn's Colitis. (2012) 6:965–90. 10.1016/j.crohns.2012.09.003 23040452

