# Supplementary material for: Galactooligosaccharide Treatment Alleviates DSS-Induced Colonic Inflammation in Caco-2 Cell Model
Source: Front Nutr. 2022 Apr 14;9:862974. doi: 10.3389/fnut.2022.862974 (PMC9047546; doi:10.3389/fnut.2022.862974)
Supplement: Supplementary file 1 [file Table_1.DOCX]

|  | **C** | **1 μg/mL Bimuno GOS** | **10 μg/mL Bimuno GOS** | **50 μg/mL Bimuno GOS** | **100 μg/mL Bimuno GOS** | **150 μg/mL Bimuno GOS** | **200 μg/mL Bimuno GOS** | **500 μg/mL Bimuno GOS** | **1000 μg/mL Bimuno GOS** | **2 % DSS** |
| --- | --- | --- | --- | --- | --- | --- | --- | --- | --- | --- |
| **C** |  | NS | NS | NS | NS | NS | NS | NS | NS | P< 0.001 |
| **1 μg/mL Bimuno GOS** |  |  | NS | NS | NS | NS | NS | NS | NS | P< 0.001 |
| **10 μg/mL Bimuno GOS** |  |  |  | NS | NS | NS | NS | NS | NS | P< 0.001 |
| **50 μg/mL Bimuno GOS** |  |  |  |  | NS | NS | NS | NS | NS | P< 0.001 |
| **100 μg/mL Bimuno GOS** |  |  |  |  |  | NS | NS | NS | NS | P< 0.001 |
| **150 μg/mL Bimuno GOS** |  |  |  |  |  |  | NS | NS | NS | P< 0.001 |
| **200 μg/mL Bimuno GOS** |  |  |  |  |  |  |  | NS | NS | P< 0.001 |
| **500 μg/mL Bimuno GOS** |  |  |  |  |  |  |  |  | NS | P< 0.001 |
| **1000 μg/mL Bimuno GOS** |  |  |  |  |  |  |  |  |  | P< 0.001 |
| **2 % DSS** |  |  |  |  |  |  |  |  |  |  |

**Table S1:** P values relative to phenol red Papp in Caco-2 cells treated with different concentrations of Bimuno GOS (Figure 2, Panel B).
