# Supplementary material for: Galactooligosaccharide Treatment Alleviates DSS-Induced Colonic Inflammation in Caco-2 Cell Model
Source: Front Nutr. 2022 Apr 14;9:862974. doi: 10.3389/fnut.2022.862974 (PMC9047546; doi:10.3389/fnut.2022.862974)
Supplement: Supplementary file 2 [file Table_2.DOCX]

|  | **C** | **0.05 % DSS** | **0.5 % DSS** | **1 % DSS** | **2 % DSS** | **3 % DSS** | **5 % DSS** |
| --- | --- | --- | --- | --- | --- | --- | --- |
| **C** |  | P< 0.05 | P< 0.05 | P< 0.01 | P< 0.001 | P< 0.001 | P< 0.001 |
| **0.05 % DSS** |  |  | NS | P< 0.05 | P< 0.01 | P< 0.001 | P< 0.001 |
| **0.5 % DSS** |  |  |  | NS | P< 0.05 | P< 0.001 | P< 0.001 |
| **1 % DSS** |  |  |  |  | P< 0.05 | P< 0.01 | P< 0.01 |
| **2 % DSS** |  |  |  |  |  | P< 0.05 | P< 0.01 |
| **3 % DSS** |  |  |  |  |  |  | NS |
| **5 % DSS** |  |  |  |  |  |  |  |

**Table S2:** P values relative to phenol red Papp in Caco-2 cells treated with different concentrations of DSS (Figure 3; Panel B).
