# Supplementary material for: Galactooligosaccharide Treatment Alleviates DSS-Induced Colonic Inflammation in Caco-2 Cell Model
Source: Front Nutr. 2022 Apr 14;9:862974. doi: 10.3389/fnut.2022.862974 (PMC9047546; doi:10.3389/fnut.2022.862974)
Supplement: Supplementary file 3 [file Table_3.DOCX]

|  | **C** | **2 % DSS + 1 μg/mL Bimuno GOS** | **2 % DSS + 10 μg/mL Bimuno GOS** | **2 % DSS + 50 μg/mL Bimuno GOS** | **2 % DSS + 100 μg/mL Bimuno GOS** | **2 % DSS + 150 μg/mL Bimuno GOS** | **2 % DSS + 200 μg/mL Bimuno GOS** | **2 % DSS + 500 μg/mL Bimuno GOS** | **2 % DSS + 1000 μg/mL Bimuno GOS** | **2 % DSS** |
| --- | --- | --- | --- | --- | --- | --- | --- | --- | --- | --- |
| **C** |  | P< 0.001 | NS | NS | NS | NS | NS | NS | NS | P< 0.01 |
| **2 % DSS + 1 μg/mL Bimuno GOS** |  |  | P< 0.001 | P< 0.001 | P< 0.001 | P< 0.001 | P< 0.001 | P< 0.001 | P< 0.001 | P< 0.05 |
| **2 % DSS + 10 μg/mL Bimuno GOS** |  |  |  | NS | NS | NS | NS | NS | NS | P< 0.001 |
| **2 % DSS + 50 μg/mL Bimuno GOS** |  |  |  | NS | NS | NS | NS | NS | NS | P< 0.001 |
| **2 % DSS + 100 μg/mL Bimuno GOS** |  |  |  |  |  | NS | NS | NS | NS | P< 0.001 |
| **2 % DSS + 150 μg/mL Bimuno GOS** |  |  |  |  |  |  | NS | NS | NS | P< 0.001 |
| **2 % DSS + 200 μg/mL Bimuno GOS** |  |  |  |  |  |  | NS | NS | NS | P< 0.001 |
| **2 % DSS + 500 μg/mL Bimuno GOS** |  |  |  |  |  |  |  |  | NS | P< 0.001 |
| **2 % DSS + 1000 μg/mL Bimuno GOS** |  |  |  |  |  |  |  |  |  | P< 0.001 |
| **2 % DSS** |  |  |  |  |  |  |  |  |  |  |

**Table S3**: P values relative to phenol red Papp in Caco-2 cells treated with 2 % DSS and different concentrations of Bimuno GOS (Figure 4 Panel B).
