# Supplementary material for: Galactooligosaccharide Treatment Alleviates DSS-Induced Colonic Inflammation in Caco-2 Cell Model
Source: Front Nutr. 2022 Apr 14;9:862974. doi: 10.3389/fnut.2022.862974 (PMC9047546; doi:10.3389/fnut.2022.862974)
Supplement: Supplementary file 4 [file Table_4.DOCX]

**Table S4a**: P values relative to differences in IL-1β secretion in Caco-2 cells treated with 2% DSS and different Bimuno GOS concentrations (Figure 5, IL-1β)

| **IL-1β** | **C** | **2 % DSS** | **1 μg/mL Bimuno GOS** | **10 μg/mL Bimuno GOS** | **50 μg/mL Bimuno GOS** | **100 μg/mL Bimuno GOS** | **200 μg/mL Bimuno GOS** | **2 % DSS + 1 μg/mL Bimuno GOS** | **2 % DSS + 10 μg/mL Bimuno GOS** | **2 % DSS + 50 μg/mL Bimuno GOS** | **2 % DSS + 100 μg/mL Bimuno GOS** | **2 % DSS + 200 μg/mL Bimuno GOS** |
| --- | --- | --- | --- | --- | --- | --- | --- | --- | --- | --- | --- | --- |
| **C** |  | P< 0.01 | NS | NS | NS | NS | NS | P< 0.05 | P< 0.05 | P< 0.05 | NS | NS |
| **2 % DSS** |  |  | P< 0.001 | P< 0.001 | P< 0.001 | P< 0.001 | P< 0.001 | P< 0.05 | P< 0.05 | P< 0.05 | P< 0.01 | P< 0.01 |
| **1 μg/mL Bimuno GOS** |  |  |  | NS | NS | NS | NS | P< 0.01 | P< 0.01 | P< 0.01 | NS | NS |
| **10 μg/mL Bimuno GOS** |  |  |  |  | NS | NS | NS | P< 0.01 | P< 0.01 | P< 0.01 | NS | NS |
| **50 μg/mL Bimuno GOS** |  |  |  |  |  | NS | NS | P< 0.05 | P< 0.01 | P< 0.01 | NS | NS |
| **100 μg/mL Bimuno GOS** |  |  |  |  |  |  | NS | P< 0.05 | P< 0.05 | P< 0.05 | NS | NS |
| **200 μg/mL Bimuno GOS** |  |  |  |  |  |  |  | P< 0.05 | P< 0.01 | P< 0.01 | NS | NS |
| **2 % DSS + 1 μg/mL Bimuno GOS** |  |  |  |  |  |  |  |  | NS | NS | P< 0.01 | P< 0.01 |
| **2 % DSS + 10 μg/mL Bimuno GOS** |  |  |  |  |  |  |  |  |  | NS | P< 0.01 | P< 0.01 |
| **2 % DSS + 50 μg/mL Bimuno GOS** |  |  |  |  |  |  |  |  |  |  | P< 0.01 | P< 0.01 |
| **2 % DSS + 100 μg/mL Bimuno GOS** |  |  |  |  |  |  |  |  |  |  |  | NS |
| **2 % DSS + 200 μg/mL Bimuno GOS** |  |  |  |  |  |  |  |  |  |  |  |  |

**Table S4b**: P values relative to differences in IL-6 secretion in Caco-2 cells treated with 2% DSS and different Bimuno GOS concentrations (Figure 5, IL-6).

| **IL-6** | **C** | **2 % DSS** | **1 μg/mL Bimuno GOS** | **10 μg/mL Bimuno GOS** | **50 μg/mL Bimuno GOS** | **100 μg/mL Bimuno GOS** | **200 μg/mL Bimuno GOS** | **2 % DSS + 1 μg/mL Bimuno GOS** | **2 % DSS + 10 μg/mL Bimuno GOS** | **2 % DSS + 50 μg/mL Bimuno GOS** | **2 % DSS + 100 μg/mL Bimuno GOS** | **2 % DSS + 200 μg/mL Bimuno GOS** |
| --- | --- | --- | --- | --- | --- | --- | --- | --- | --- | --- | --- | --- |
| **C** |  | P< 0.01 | NS | NS | NS | NS | NS | P< 0.01 | P< 0.01 | P< 0.01 | NS | NS |
| **2% DSS** |  |  | P< 0.01 | P< 0.01 | P< 0.01 | P< 0.01 | P< 0.01 | NS | NS | NS | P< 0.01 | P< 0.01 |
| **1 μg/mL Bimuno GOS** |  |  |  | NS | NS | NS | NS | P< 0.01 | P< 0.01 | P< 0.01 | NS | NS |
| **10 μg/mL Bimuno GOS** |  |  |  |  | NS | NS | NS | P< 0.05 | P< 0.05 | P< 0.05 | NS | NS |
| **50 μg/mL Bimuno GOS** |  |  |  |  |  | NS | NS | P< 0.01 | P< 0.01 | P< 0.01 | NS | NS |
| **100 μg/mL Bimuno GOS** |  |  |  |  |  |  | NS | P< 0.01 | P< 0.01 | P< 0.01 | NS | NS |
| **200 μg/mL Bimuno GOS** |  |  |  |  |  |  |  | P< 0.01 | P< 0.01 | P< 0.01 | NS | NS |
| **2 % DSS + 1 μg/mL Bimuno GOS** |  |  |  |  |  |  |  |  | NS | NS | P< 0.01 | P< 0.01 |
| **2 % DSS + 10 μg/mL Bimuno GOS** |  |  |  |  |  |  |  |  |  | NS | P< 0.01 | P< 0.01 |
| **2 % DSS + 50 μg/mL Bimuno GOS** |  |  |  |  |  |  |  |  |  |  | P< 0.01 | P< 0.01 |
| **2 % DSS + 100 μg/mL Bimuno GOS** |  |  |  |  |  |  |  |  |  |  |  | P< 0.01 |
| **2 % DSS + 200 μg/mL Bimuno GOS** |  |  |  |  |  |  |  |  |  |  |  |  |

**Table S4c**: P values relative to differences in IL-8 secretion in Caco-2 cells treated with 2% DSS and different Bimuno GOS concentrations (Figure 5, IL-8).

| **IL-8** | **C** | **2 % DSS** | **1 μg/mL Bimuno GOS** | **10 μg/mL Bimuno GOS** | **50 μg/mL Bimuno GOS** | **100 μg/mL Bimuno GOS** | **200 μg/mL Bimuno GOS** | **2 % DSS + 1 μg/mL Bimuno GOS** | **2 % DSS + 10 μg/mL Bimuno GOS** | **2 % DSS + 50 μg/mL Bimuno GOS** | **2 % DSS + 100 μg/mL Bimuno GOS** | **2 % DSS + 200 μg/mL Bimuno GOS** |
| --- | --- | --- | --- | --- | --- | --- | --- | --- | --- | --- | --- | --- |
| **C** |  | P< 0.01 | NS | NS | NS | NS | NS | P< 0.01 | P< 0.01 | P< 0.01 | NS | NS |
| **2% DSS** |  |  | P< 0.01 | P< 0.01 | P< 0.01 | P< 0.01 | P< 0.01 | NS | NS | NS | P< 0.01 | P< 0.01 |
| **1 μg/mL Bimuno GOS** |  |  |  | NS | NS | NS | NS | P< 0.01 | P< 0.01 | P< 0.01 | NS | NS |
| **10 μg/mL Bimuno GOS** |  |  |  |  | NS | NS | NS | P< 0.05 | P< 0.05 | P< 0.05 | NS | NS |
| **50 μg/mL Bimuno GOS** |  |  |  |  |  | NS | NS | P< 0.01 | P< 0.01 | P< 0.01 | NS | NS |
| **100 μg/mL Bimuno GOS** |  |  |  |  |  |  | NS | P< 0.01 | P< 0.01 | P< 0.01 | NS | NS |
| **200 μg/mL Bimuno GOS** |  |  |  |  |  |  |  | P< 0.01 | P< 0.01 | P< 0.01 | NS | NS |
| **2 % DSS + 1 μg/mL Bimuno GOS** |  |  |  |  |  |  |  |  | NS | NS | P< 0.01 | P< 0.01 |
| **2 % DSS + 10 μg/mL Bimuno GOS** |  |  |  |  |  |  |  |  |  | NS | P< 0.01 | P< 0.01 |
| **2 % DSS + 50 μg/mL Bimuno GOS** |  |  |  |  |  |  |  |  |  |  | P< 0.01 | P< 0.01 |
| **2 % DSS + 100 μg/mL Bimuno GOS** |  |  |  |  |  |  |  |  |  |  |  | P< 0.01 |
| **2 % DSS + 200 μg/mL Bimuno GOS** |  |  |  |  |  |  |  |  |  |  |  |  |

**Table S4d**: P values relative to differences in TNF-α secretion in Caco-2 cells treated with 2% DSS and different Bimuno GOS concentrations (Figure 5, TNF-α).

| **TNF-α** | **C** | **2 % DSS** | **1 μg/mL Bimuno GOS** | **10 μg/mL Bimuno GOS** | **50 μg/mL Bimuno GOS** | **100 μg/mL Bimuno GOS** | **200 μg/mL Bimuno GOS** | **2 % DSS + 1 μg/mL Bimuno GOS** | **2 % DSS + 10 μg/mL Bimuno GOS** | **2 % DSS + 50 μg/mL Bimuno GOS** | **2 % DSS + 100 μg/mL Bimuno GOS** | **2 % DSS + 200 μg/mL Bimuno GOS** |
| --- | --- | --- | --- | --- | --- | --- | --- | --- | --- | --- | --- | --- |
| **C** |  | P< 0.01 | NS | NS | NS | NS | NS | P< 0.01 | P< 0.01 | P< 0.05 | NS | NS |
| **2% DSS** |  |  | P< 0.01 | P< 0.01 | P< 0.01 | P< 0.01 | P< 0.01 | NS | P< 0.05 | P< 0.05 | P< 0.01 | P< 0.01 |
| **1 μg/mL Bimuno GOS** |  |  |  | NS | NS | NS | NS | P< 0.01 | P< 0.01 | P< 0.05 | NS | NS |
| **10 μg/mL Bimuno GOS** |  |  |  |  | NS | NS | NS | P< 0.01 | P< 0.01 | P< 0.05 | NS | NS |
| **50 μg/mL Bimuno GOS** |  |  |  |  |  | NS | NS | P< 0.01 | P< 0.01 | P< 0.05 | NS | NS |
| **100 μg/mL Bimuno GOS** |  |  |  |  |  |  | NS | P< 0.01 | P< 0.01 | P< 0.05 | NS | NS |
| **200 μg/mL Bimuno GOS** |  |  |  |  |  |  |  | P< 0.01 | P< 0.01 | P< 0.01 | NS | NS |
| **2 % DSS + 1 μg/mL Bimuno GOS** |  |  |  |  |  |  |  |  | P< 0.05 | P< 0.01 | P< 0.01 | P< 0.01 |
| **2 % DSS + 10 μg/mL Bimuno GOS** |  |  |  |  |  |  |  |  |  | P< 0.05 | P< 0.01 | P< 0.01 |
| **2 % DSS + 50 μg/mL Bimuno GOS** |  |  |  |  |  |  |  |  |  |  | P< 0.05 | P< 0.05 |
| **2 % DSS + 100 μg/mL Bimuno GOS** |  |  |  |  |  |  |  |  |  |  |  | NS |
| **2 % DSS + 200 μg/mL Bimuno GOS** |  |  |  |  |  |  |  |  |  |  |  |  |
