# Supplementary material for: Galactooligosaccharide Treatment Alleviates DSS-Induced Colonic Inflammation in Caco-2 Cell Model
Source: Front Nutr. 2022 Apr 14;9:862974. doi: 10.3389/fnut.2022.862974 (PMC9047546; doi:10.3389/fnut.2022.862974)
Supplement: Supplementary file 5 [file Table_5.DOCX]

**Table S5a**: P values relative to differences in TLR4 protein expression in Caco-2 cells treated with 2 % DSS and 100 or 200 µg/mL Bimuno GOS (Figure 6, TLR4).

| **TLR4** | **C** | **2 % DSS** | **100 μg/mL Bimuno GOS** | **200 μg/mL Bimuno GOS** | **2 % DSS + 100 μg/mL Bimuno GOS** | **2 % DSS + 200 μg/mL Bimuno GOS** |
| --- | --- | --- | --- | --- | --- | --- |
| **C** |  | P< 0.05 | P< 0.01 | P< 0.01 | P< 0.01 | P< 0.05 |
| **2 % DSS** |  |  | P< 0.01 | P< 0.01 | P< 0.01 | P< 0.05 |
| **100 μg/mL Bimuno GOS** |  |  |  | NS | NS | NS |
| **200 μg/mL Bimuno GOS** |  |  |  |  | NS | NS |
| **2 % DSS + 100 μg/mL Bimuno GOS** |  |  |  |  |  | NS |
| **2 % DSS + 200 μg/mL Bimuno GOS** |  |  |  |  |  |  |

**Table S5b**: P values relative to differences in MyD88 protein expression in Caco-2 cells treated with 2 % DSS and 100 or 200 µg/mL Bimuno GOS (Figure 6, MyD88).

| **MyD88** | **C** | **2 % DSS** | **100 μg/mL Bimuno GOS** | **200 μg/mL Bimuno GOS** | **2 % DSS + 100 μg/mL Bimuno GOS** | **2 % DSS + 200 μg/mL Bimuno GOS** |
| --- | --- | --- | --- | --- | --- | --- |
| **C** |  | P< 0.05 | NS | NS | NS | NS |
| **2 % DSS** |  |  | P< 0.01 | P< 0.01 | P< 0.01 | P< 0.05 |
| **100 μg/mL Bimuno GOS** |  |  |  | NS | NS | NS |
| **200 μg/mL Bimuno GOS** |  |  |  |  | NS | NS |
| **2 % DSS + 100 μg/mL Bimuno GOS** |  |  |  |  |  | NS |
| **2 % DSS + 200 μg/mL Bimuno GOS** |  |  |  |  |  |  |

**Table S5c**: P values relative to differences in P-IKKα/IKKα expression in Caco-2 cells treated with 2 % DSS and 100 or 200 µg/mL Bimuno GOS (Figure 6, P-IKKα/IKKα).

| **P-IKKα/IKKα** | **C** | **2 % DSS** | **100 μg/mL Bimuno GOS** | **200 μg/mL Bimuno GOS** | **2 % DSS + 100 μg/mL Bimuno GOS** | **2 % DSS + 200 μg/mL Bimuno GOS** |
| --- | --- | --- | --- | --- | --- | --- |
| **C** |  | P< 0.05 | NS | NS | NS | NS |
| **2 % DSS** |  |  | P< 0.05 | P< 0.05 | P< 0.05 | P< 0.05 |
| **100 μg/mL Bimuno GOS** |  |  |  | NS | NS | NS |
| **200 μg/mL Bimuno GOS** |  |  |  |  | NS | NS |
| **2 % DSS + 100 μg/mL Bimuno GOS** |  |  |  |  |  | NS |
| **2 % DSS + 200 μg/mL Bimuno GOS** |  |  |  |  |  |  |

**Table S5d**: P values relative to differences in P-IKKβ/IKKβ expression in Caco-2 cells treated with 2 % DSS and 100 or 200 µg/mL Bimuno GOS (Figure 6, P-IKKβ/IKKβ).

| **P-IKKβ/IKKβ** | **C** | **2 % DSS** | **100 μg/mL Bimuno GOS** | **200 μg/mL Bimuno GOS** | **2 % DSS + 100 μg/mL Bimuno GOS** | **2 % DSS + 200 μg/mL Bimuno GOS** |
| --- | --- | --- | --- | --- | --- | --- |
| **C** |  | P< 0.05 | NS | NS | NS | NS |
| **2 % DSS** |  |  | P< 0.05 | P< 0.05 | P< 0.05 | P< 0.05 |
| **100 μg/mL Bimuno GOS** |  |  |  | NS | NS | NS |
| **200 μg/mL Bimuno GOS** |  |  |  |  | NS | NS |
| **2 % DSS + 100 μg/mL Bimuno GOS** |  |  |  |  |  | NS |
| **2 % DSS + 200 μg/mL Bimuno GOS** |  |  |  |  |  |  |

**Table S5e**: P values relative to differences in P-IKbα/IKbα expression in Caco-2 cells treated with 2 % DSS and 100 or 200 µg/mL Bimuno GOS (Figure 6, P-IKbα/ IKbα).

| **P-IKbα/ IKbα** | **C** | **2 % DSS** | **100 μg/mL Bimuno GOS** | **200 μg/mL Bimuno GOS** | **2 % DSS + 100 μg/mL Bimuno GOS** | **2 % DSS + 200 μg/mL Bimuno GOS** |
| --- | --- | --- | --- | --- | --- | --- |
| **C** |  | P< 0.01 | NS | NS | NS | NS |
| **2 % DSS** |  |  | P< 0.01 | P< 0.05 | P< 0.01 | P< 0.01 |
| **100 μg/mL Bimuno GOS** |  |  |  | NS | NS | NS |
| **200 μg/mL Bimuno GOS** |  |  |  |  | NS | NS |
| **2 % DSS + 100 μg/mL Bimuno GOS** |  |  |  |  |  | NS |
| **2 % DSS + 200 μg/mL Bimuno GOS** |  |  |  |  |  |  |

**Table S5f**: P values relative to differences in P-p65/p65 expression in Caco-2 cells treated with 2 % DSS and 100 or 200 µg/mL Bimuno GOS (Figure 6, P-p65/p65).

| **P-p65/p65** | **C** | **2 % DSS** | **100 μg/mL Bimuno GOS** | **200 μg/mL Bimuno GOS** | **2 % DSS + 100 μg/mL Bimuno GOS** | **2 % DSS + 200 μg/mL Bimuno GOS** |
| --- | --- | --- | --- | --- | --- | --- |
| **C** |  | P< 0.01 | NS | NS | NS | NS |
| **2 % DSS** |  |  | P< 0.01 | P< 0.05 | P< 0.01 | P< 0.01 |
| **100 μg/mL Bimuno GOS** |  |  |  | NS | NS | NS |
| **200 μg/mL Bimuno GOS** |  |  |  |  | NS | NS |
| **2 % DSS + 100 μg/mL Bimuno GOS** |  |  |  |  |  | NS |
| **2 % DSS + 200 μg/mL Bimuno GOS** |  |  |  |  |  |  |

**Table S5g**: P values relative to differences in Tollip protein expression in Caco-2 cells treated with 2 % DSS and 100 or 200 µg/mL Bimuno GOS (Figure 6, Tollip).

| **Tollip** | **C** | **2 % DSS** | **100 μg/mL Bimuno GOS** | **200 μg/mL Bimuno GOS** | **2 % DSS + 100 μg/mL Bimuno GOS** | **2 % DSS + 200 μg/mL Bimuno GOS** |
| --- | --- | --- | --- | --- | --- | --- |
| **C** |  | P< 0.01 | NS | NS | NS | NS |
| **2 % DSS** |  |  | P< 0.01 | P< 0.01 | P< 0.01 | P< 0.01 |
| **100 μg/mL Bimuno GOS** |  |  |  | NS | NS | NS |
| **200 μg/mL Bimuno GOS** |  |  |  |  | NS | NS |
| **2 % DSS + 100 μg/mL Bimuno GOS** |  |  |  |  |  | NS |
| **2 % DSS + 200 μg/mL Bimuno GOS** |  |  |  |  |  |  |

**Table S5h**: P values relative to differences in IRAK-M protein expression in Caco-2 cells treated with 2 % DSS and 100 or 200 µg/mL Bimuno GOS (Figure 6, IRAK-M).

| **IRAK-M** | **C** | **2 % DSS** | **100 μg/mL Bimuno GOS** | **200 μg/mL Bimuno GOS** | **2 % DSS + 100 μg/mL Bimuno GOS** | **2 % DSS + 200 μg/mL Bimuno GOS** |
| --- | --- | --- | --- | --- | --- | --- |
| **C** |  | P< 0.01 | NS | NS | NS | NS |
| **2 % DSS** |  |  | P< 0.01 | P< 0.01 | P< 0.01 | P< 0.01 |
| **100 μg/mL Bimuno GOS** |  |  |  | NS | NS | NS |
| **200 μg/mL Bimuno GOS** |  |  |  |  | NS | NS |
| **2 % DSS + 100 μg/mL Bimuno GOS** |  |  |  |  |  | NS |
| **2 % DSS + 200 μg/mL Bimuno GOS** |  |  |  |  |  |  |
